# Testing the Translational Power of the Zebrafish: An Interspecies Analysis of Responses to Cardiovascular Drugs

**DOI:** 10.3389/fphar.2019.00893

**Published:** 2019-08-16

**Authors:** Luigi Margiotta-Casaluci, Stewart F. Owen, Mariann Rand-Weaver, Matthew J. Winter

**Affiliations:** ^1^College of Health and Life Sciences, Brunel University London, London, United Kingdom; ^2^Global Safety, Health & Environment, AstraZeneca, Alderley Park, Macclesfield, United Kingdom; ^3^School of Biosciences, College of Life and Environmental Science, University of Exeter, Exeter, United Kingdom

**Keywords:** drug safety, cardiovascular effects, zebrafish, preclinical species, meta-analysis, comparative pharmacology, beta-adrenergic receptor, renin–angiotensin system

## Abstract

The zebrafish is rapidly emerging as a promising alternative *in vivo* model for the detection of drug-induced cardiovascular effects. Despite its increasing popularity, the ability of this model to inform the drug development process is often limited by the uncertainties around the quantitative relevance of zebrafish responses compared with nonclinical mammalian species and ultimately humans. In this test of concept study, we provide a comparative quantitative analysis of the *in vivo* cardiovascular responses of zebrafish, rat, dog, and human to three model compounds (propranolol, losartan, and captopril), which act as modulators of two key systems (beta-adrenergic and renin–angiotensin systems) involved in the regulation of cardiovascular functions. We used *in vivo* imaging techniques to generate novel experimental data of drug-mediated cardiovascular effects in zebrafish larvae. These data were combined with a database of interspecies mammalian responses (i.e., heart rate, blood flow, vessel diameter, and stroke volume) extracted from the literature to perform a meta-analysis of effect size and direction across multiple species. In spite of the high heterogeneity of study design parameters, our analysis highlighted that zebrafish and human responses were largely comparable in >80% of drug/endpoint combinations. However, it also revealed a high intraspecies variability, which, in some cases, prevented a conclusive interpretation of the drug-induced effect. Despite the shortcomings of our study, the meta-analysis approach, combined with a suitable data visualization strategy, enabled us to observe patterns of response that would likely remain undetected with more traditional methods of qualitative comparative analysis. We propose that expanding this approach to larger datasets encompassing multiple drugs and modes of action would enable a rigorous and systematic assessment of the applicability domain of the zebrafish from both a mechanistic and phenotypic standpoint. This will increase the confidence in its application for the early detection of adverse drug reactions in any major organ system.

## Introduction

A considerable number of drug candidates have the potential to alter cardiovascular functions at therapeutically relevant concentrations. Predicting those effects as early as possible during drug development is critically important to ensure the progression of safer compounds through the pipeline and to minimize the risk of cardiovascular safety liabilities emerging at later stages of development (Laverty et al., 2011; Cook et al., 2014; Lester and Olbertz, 2016). The fast-paced advancements ongoing in the development of human-based *in silico* and *in vitro* predictive approaches hold great promise for improving the early detection of drug-induced cardiovascular alterations, including cardiotoxicity (Clements et al., 2015; Colatsky et al., 2016; Gintant et al., 2016; Land et al., 2017; Passini et al., 2017). However, to date, the use of *in vivo* preclinical models is still a key aspect of cardiovascular efficacy and safety assessment (Fliegner et al., 2015; Vargas et al., 2015; Berridge et al., 2016), mainly because of the ability of *in vivo* testing to capture integrated multiscale processes that cannot be observed outside an intact organism. These processes include pharmacokinetic-dependent and metabolism-mediated effects, chronic or delayed toxicity, vascular and hemodynamic alterations, as well as interaction between cardiovascular, nervous, and renal systems (Holzgrefe et al., 2014).

In this context, the identification of the most suitable preclinical animal model represents a central challenge for the design of a successful testing strategy, as this choice can profoundly affect the translational value of each experiment and, in turn, data interpretation and subsequent decision-making (Denayer et al., 2014; Holzgrefe et al., 2014). From a cardiovascular perspective, dog and nonhuman primates (e.g., cynomolgus monkey) are the most commonly used nonrodent models, as their physiology is considered the most relevant to humans (Leishman et al., 2012; Holzgrefe et al., 2014). Other test species include minipig (Bode et al., 2010), marmoset (Tabo et al., 2008), and guinea pigs (Marks et al., 2012). Beside these models, small rodent species (i.e., rat and mouse) remain the most popular choice to investigate cardiovascular physiology and disease, genetics, and pharmacology (Camacho et al., 2016). As with any animal model, each species mentioned above has both advantages and limitations (e.g., see Holzgrefe et al. (2014) and Milani-Nejad and Janssen (2014) for extensive reviews of these aspects); however, common limitations include high ethical and financial costs, and low throughput potential.

In recent years, extensive research efforts have been allocated worldwide to identify potential alternative testing approaches that may lead to the reduction, replacement, or refinement (3Rs) of the model species mentioned above. Within this research theme, the zebrafish has emerged as a new, potentially valuable, model for the *in vivo* assessment of a variety of human-relevant drug-induced effects, including cardiovascular alterations (Parker et al., 2014; MacRae and Peterson, 2015). Zebrafish are characterized by a number of valuable features, including relatively inexpensive maintenance costs, amenability to genetic manipulation, high conservation of human drug targets (i.e., >82%; Howe et al., 2013; Verbruggen et al., 2017), and of a broad range of human-relevant phenotypes that can be modified by pharmacological treatment (MacRae and Peterson, 2015).

Considering the high impact of unpredicted cardiotoxicity on drug development (Laverty et al., 2011), the availability of a simpler vertebrate model, such as zebrafish, may enable cardiovascular profiling of new drugs before commencing mammalian toxicity tests, thus serving as a bridge between early *in vitro* safety predictions and later *in vivo* preclinical testing. Several studies have started to explore this potential from a translational perspective, such as Parker et al. (2014)and Cornet et al. (2017). Despite encouraging results, to date, the implementation of zebrafish in existing testing strategies faces resistance not least because of uncertainty around the quantitative aspects of zebrafish cardiovascular responses compared with both mammalian preclinical species and humans. We propose that coordinated efforts to perform quantitative comparative assessment of those responses may help to clarify the translational value of zebrafish and help define its domain of applicability from both mechanistic and phenotypic standpoints.

**Figure 1:**
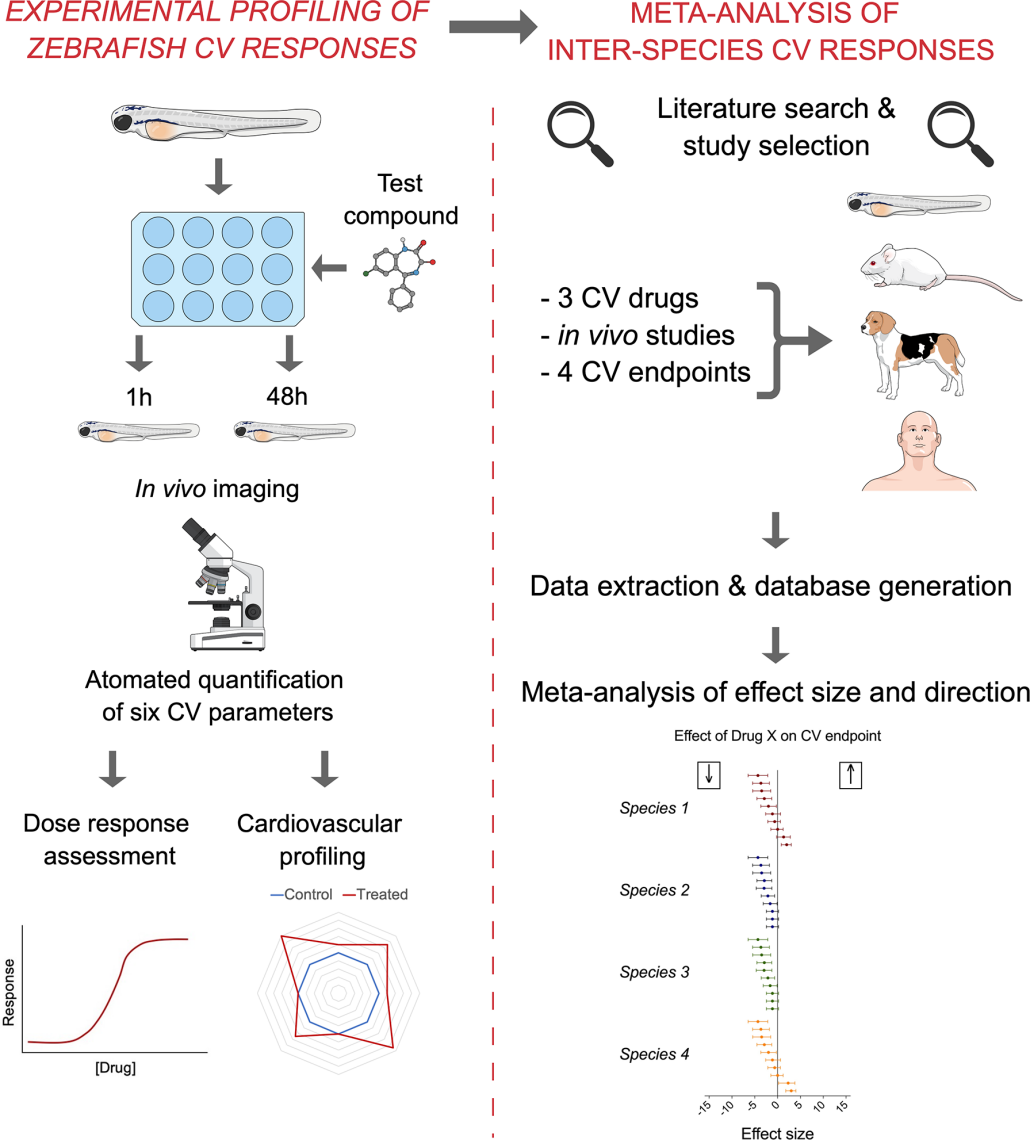
Summary of the methodological approach employed in the study. The experimental quantification of zebrafish (7 dpf) cardiovascular responses to propranolol, losartan, and captopril (left) was combined with the subsequent meta-analysis of mammalian preclinical and human data extracted from the literature (right) to generate quantitative understanding of interspecies similarities in effect size and direction.

The aim of the present study was to quantify the degree of similarity in the *in vivo* cardiovascular responses of zebrafish, rat, dog, and human to three model compounds (propranolol, losartan, and captopril), which act as modulators of two key systems (beta-adrenergic and renin–angiotensin systems) involved in the regulation of cardiovascular functions. To do so, we used *in vivo* imaging techniques to generate novel zebrafish experimental data. The latter were successively combined with a database of interspecies responses extracted from the literature to perform a meta-analysis of effect size and direction across species (**Figure 1**).

## Materials and Methods

### Experimental Animal Culture

Adult WIK-strain (wild-type India Kolkata) zebrafish were maintained in flow through aquaria under optimal spawning conditions. Embryos were collected from individual male–female pairs and cultured in Petri dishes to 7 days postfertilization (dpf), as described in Winter et al. (2008). A complete water change was carried out every 24 h ensuring that water quality was maintained until day 7, when the fish were used in the experiments. All experiments were conducted in temperature-controlled laboratories held at 28 ± 1°C. Animals were treated in full accordance with the United Kingdom Animals (Scientific Procedures) Act regarding the use of animals in scientific procedures. All sections of this report adhere to the ARRIVE Guidelines for reporting animal research (Kilkenny et al., 2010). A completed ARRIVE guidelines checklist is included in the **Supplementary Material Data Sheet 2**.

### Test Compounds and Reagents

All test compounds and reagents used were purchased from Sigma-Aldrich UK Ltd. Propranolol (CAS no. 318-98-9), losartan (CAS no. 124750-99-8), and captopril (CAS number 62571-86-2) were selected as model compounds because of their known pharmacological activity (respectively, beta-adrenergic receptor antagonist, angiotensin 2 receptor antagonist, and angiotensin-converting enzyme inhibitor) and for the public availability of preclinical data specifically relating to their effects on the cardiovascular system.

### Determination of Maximum Tolerated Concentration (MTC)

Individual larvae were loaded into each well of 24-well microplates in a total volume of 500 μl of dechlorinated tap water (culture water). To determine the maximum tolerated concentration (MTC), each test compound was tested at seven different concentrations using eight zebrafish larvae per treatment group, in parallel with a solvent control group [0.5–1% (*v/v*) DMSO]. Test compounds were freshly prepared in 2% (*v/v*) DMSO in culture water, and the pH of stock test compound and controls was checked and adjusted to 7.4 using 1 M NaOH/1 M HCl, before subsequent dilution. The allocation of each exposure concentration to specific columns of the multiwell plate was randomized, as well as the allocation of individual zebrafish to individual wells. After 1 h, the MTC was defined using a series of qualitative indicators of animal health as previously outlined in Winter et al. (2008). Briefly, these were loss of dorso-ventral balance, abnormal morphology, larval touch responsiveness using a seeker, and mortality indicated by the absence of heartbeat.

### Drug Administration for CV Assessment

The assessment of cardiovascular function was performed as previously described by Parker et al. (2014). Individual larvae were loaded into each well of 24-well microplates in a total volume of 500 μl of culture water. Each compound was tested at four different concentrations using six larvae per treatment. Each experiment also included a solvent control group (0.5–1% DMSO) with the same number of larvae. The selection of the concentration range used to assess dose responsiveness was driven by the MTC data so that the highest nonlethal concentration was used as the apical concentration in the final concentration-response experiments. The allocation of each exposure concentration to specific columns of the multiwell plate was randomized, as well as the allocation of individual zebrafish to individual wells. Two sets of independent experiments were performed to quantify drug-induced effects after 1 and 48 h exposure. In the 1-h exposure experiments, propranolol was tested at 16, 32, 64, and 125 µM; losartan was tested at 1.25, 2.5. 5, and 10 mM; captopril was tested at 6.25, 12.5, 25, and 50 mM. In the 48-h exposure experiment, propranolol was tested at 2, 4, 8, and 16 µM; losartan was tested at 0.625, 1.25, 2.5, and 5 mM; captopril was tested at 6.25, 12.5, 25, and 50 mM. Larvae were dosed by immersion at 30-min intervals so that they could be mounted individually, and cardiovascular function was assessed for 20 min. Each compound was tested over 2 days; on each day, three fish were assessed for each treatment group (*n* = 6). This design required the use of two different clutches of fish to minimize the risk of bias associated with clutch-specific sensitivity.

### Preparation of Animals and Video Capture

As previously stated, the detailed methodology used for the *in vivo* quantification of cardiovascular function is identical to that described by Parker et al. (2014). Briefly, following drug exposure, each larva was anesthetized with 0.1 mg/ml MS222 (pH 7.5) until dorso-ventral balance was lost, rapidly transferred into low melting point agarose (10 mg/ml, held as a liquid at 35°C), and then deposited in a total volume of 80 μl into a single well created by a press-to-seal silicon isolator (Sigma-Aldrich, Poole, UK) on a clear microscope slide. The orientation of the larva was gently adjusted to offer a lateral view with its head to the left. To maintain the position, the agarose was rapidly solidified by a brief exposure to a cooling plate set at 5°C. Two drops of MS222 were placed on top, followed by a coverslip to minimize evaporation and gel contraction. The slide was then transferred to an inverted light microscope (Leica DM IRB, Leica Microsystems UK Ltd., 5X objective) fitted with two high-speed video cameras. One camera was positioned to capture the whole heart at 30 frames per second (fps) (Grasshopper^®^ GRAS-50S5C-C) and the second to capture the dorsal aorta, caudal to the swim bladder, at 120 fps (Grasshopper^®^ GRAS-03K2M-C). Both cameras were independently focused on their respective regions of interest to ensure optimal image quality and set to record simultaneously for 20 min.

### Analysis of Cardiac and Vascular Parameters

Heart videos were analyzed using MicroZebraLab™ (v3.5, ViewPoint, Lyon, France). The software provides beat frequencies for each chamber, allowing the determination of the global heart rate (atrial and ventricular beat rates per minute or ABR and VBR, respectively), as well as the detection of potential arrhythmias (e.g., A–V decoupling) *via* the quantification of atrium–ventriculum beat ratio (A–V beat ratio). Blood flow videos were analyzed using ZebraBlood™ (v1.3.2, ViewPoint, Lyon, France), which enabled quantification of changes in blood vessel diameter (i.e., dorsal aorta diameter, DA diameter) and dorsal aorta blood flowrate (DA flow), as described by Parker et al. (2014). A surrogate measure of cardiac stroke volume (surrogate stroke volume, SSV) was calculated by dividing the dorsal aorta flow rate (in nl/s) by the VBR per second (bpm/60), as also previously described in Parker et al. (2014).

### Analysis of Zebrafish Cardiovascular Data

Statistical analyses were conducted using GraphPad Prism 7 software. Data were analyzed for normality (Kolmogorov–Smirnov test) and homogeneity of variance (Levene’s test). Where the assumptions for parametric testing were met, one-way analysis of variance (ANOVA) was undertaken, followed by the Dunnett’s test to compare the treatment means with respective controls. Where the assumptions were not met, data were analyzed using a Kruskal–Wallis ANOVA on Ranks, followed by Dunn’s *post hoc* test. Power analysis was performed using the Experimental Design Assistant (EDA) online tool operated by the UK National Centre for the Replacement, Refinement and Reduction of Animals in Research (NC3Rs) (https://eda.nc3rs.org.uk) (Sert et al., 2017) to estimate the endpoint-specific minimum effect size likely to be detected with *n* = 6. The latter was calculated using endpoint-specific mean and standard deviation observed in the control populations, with power set at 0.80 and significance level (alpha) set at 0.05 (two-sided test).

### Meta-Analysis

The objective of the meta-analysis was to estimate drug-induced effect size and effect direction (increase or decrease) across four different species (zebrafish, rat, dog, and human) using data collected from publicly available literature.

#### Data Sources and Literature Search

A literature search *via* PubMed and Google Scholar was performed between January 2017 and January 2018 to identify experimental studies that quantified the effects of propranolol, losartan, and captopril on four cardiovascular parameters (heart rate, blood flow, stroke volume, and blood vessel diameter) in four species (zebrafish, dog, rat, and human). The search was performed using a combination of keywords (drug name + endpoint name + species) and was restricted to English language publications only. Studies were included if they reported quantification of one or more of the target endpoints in both control and drug-treated subjects. Specifically, the minimum amount of information necessary for inclusion consisted of number of experimental subjects, mean value, and standard deviation, in both control and treated groups.

#### Data Extraction and Database Quality Assessment

The data and information extracted from each relevant publication included species, study design, sample size of control group, sample size of treatment group, mean value and standard deviation of control group, mean value and standard deviation of treatment group, dose, administration route, treatment duration, and pathological status of the experimental subjects (e.g., healthy vs. disease models). For the studies that reported dose and/or time responses, each dose and/or time point was considered an independent data point in the subsequent meta-analysis. A quality assessment of all extracted data and relative database was performed to evaluate the consistency between extracted data and original values. All identified inconsistencies were resolved before the final analysis. The zebrafish data generated here, in our zebrafish experiments, were also included in the database.

#### Data Synthesis and Statistical Analysis

Extracted data were combined for meta-analysis using Open Meta-Analyst software (by Center of Evidence Based Medicine http://www.cebm.brown.edu/openmeta/). For each endpoint, subgroup meta-analyses were conducted using a random effect model according to the DerSimonian–Laird method (DerSimonian and Laird, 1986). Each species represented a subgroup. Forest plots were generated to summarize the effect size estimates (expressed as standardized mean differences, SMD) and their 95% confidence intervals for each of the four species. These figures include measures of heterogeneity across studies (*I* ^2^ statistic) and a test for overall effect.

## Results

### Drug-Induced Cardiovascular Effects in Zebrafish

**Figure 2:**
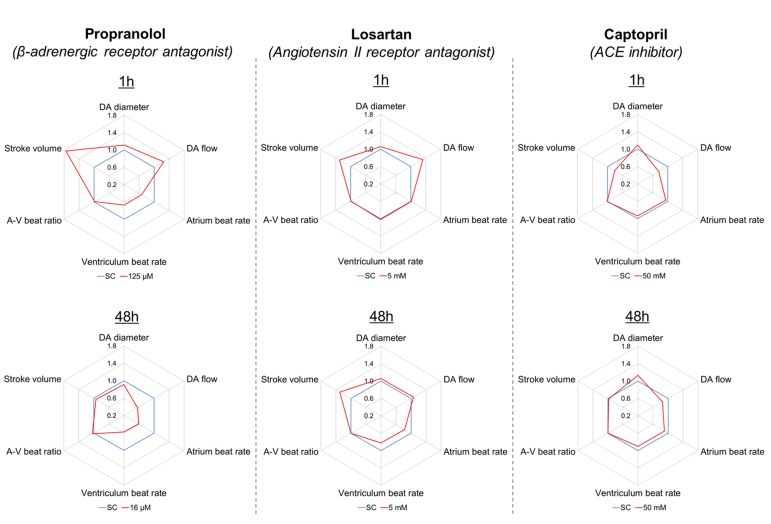
Integrated representation of the effects induced by propranolol (125 µM), losartan (5 mM), and captopril (50 mM) on six cardiovascular endpoints measured in zebrafish larvae (7 dpf), after 1 and 48 h exposure. Each graph represents the effect size observed for each endpoint in treated fish (red) versus control fish (blue), expressed as ratio between mean treated value and mean control value. For example, a treated value of 1.1 indicates a 10% increase versus the control value.

**Figure 3:**
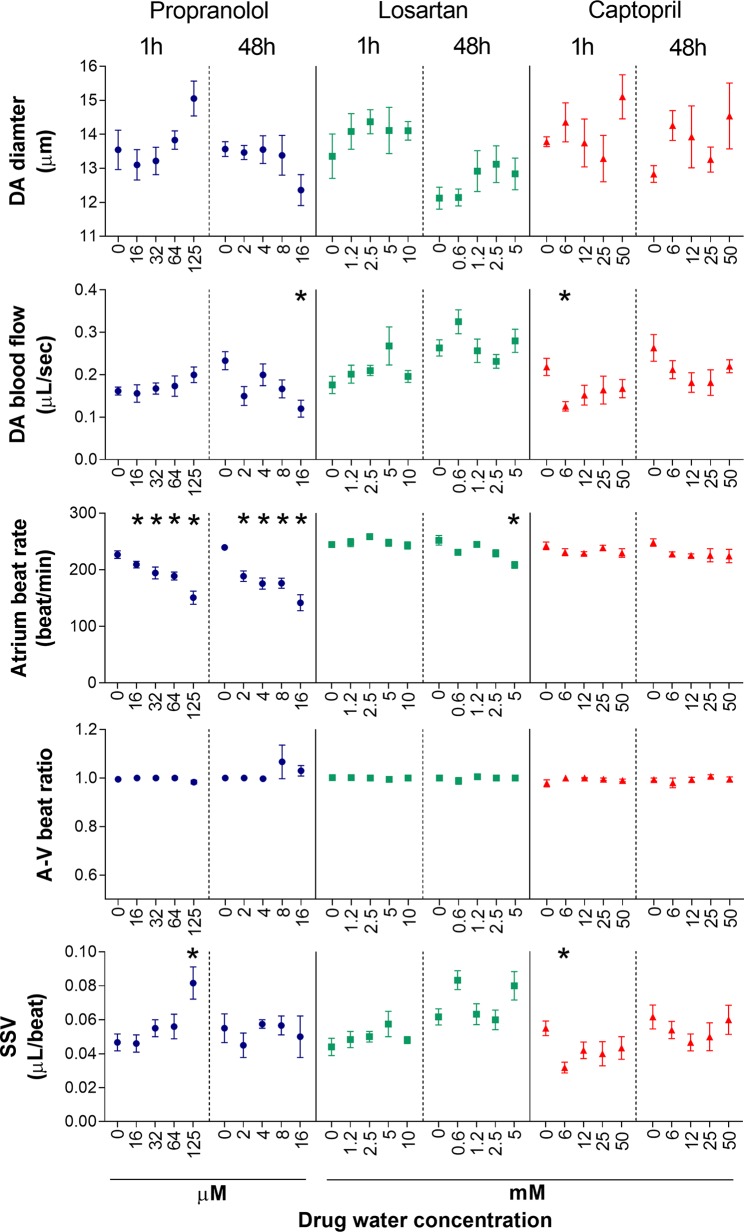
Dose–response of five cardiovascular parameters measured in zebrafish larvae (7 dpf) following exposure to propranolol, losartan, and captopril after two different exposure times (1 and 48 h). Data are presented as mean ± SEM (*n* = 4–6). Statistically significant differences from the control group are displayed as *(*p* < 0.05).

To test the effects of the three model drugs on zebrafish cardiovascular functions, we used *in vivo* imaging to quantify the response of six cardiovascular parameters after 1 and 48 h of exposure. A visualization of the six endpoints as integrated cardiovascular functional outputs is displayed in **Figure 2**, whereas **Figure 3** shows the entire set of *in vivo* data generated during the study. Considering the interdependence of the cardiovascular parameters considered in this study, the double visualization strategy of the same data allowed us to evaluate both individual endpoints and the integrated cardiovascular responses to the drug, facilitating the interpretation of the data and the detection of shifts from average control physiology. This profiling approach also enabled a more effective interdrug comparison. Mean, minimum, and maximum values quantified in each experimental group are summarized in the **Supplementary Tables 1-6** (**Supplementary Material Data Sheet 1**). The estimated endpoint-specific minimum effect size values (%) likely to be detected with *n* = 6, for each endpoint/drug/time combination, are provided in **Supplementary Table 7** (**Supplementary Material Data Sheet 1**). In the following sections, we describe, in detail, the effects of each compound on the cardiovascular system of each of the model species evaluated. It is worth noting that, in addition to effects showing statistical significance, we have also included some discussion of nonstatistically significant effects that, in the context of our meta-analysis, were considered to be of biological importance. Our justification for this approach is that we hypothesized that even small (for example, 10–20%) changes in the cardiovascular parameters considered here are likely to have high biological impact, for example a positive therapeutic effect for the patient (Cucherat, 2007; Leucht et al., 2015). This is an ongoing issue with the interpretation of data from animal studies in which relatively small numbers of test subjects are typically employed, in contrast to the need for large-scale clinical trials to demonstrate, in many cases, what are relatively small therapeutic advantages.

#### Propranolol

Fish exposed to propranolol for 1 h (**Figure 2**, **Figure 3**) displayed a clear dose-dependent decrease in both ABR and VBR, and the effect was statistically significant at the two highest drug concentrations (64 and 125 µM: at 64 µM, ABR: −17 ± 7%, *p* = 0.014; VBR: −17 ± 7%, *p* = 0.047; at 125 µM, ABR: −33 ± 13%, *p* < 0.0001; VBR: −33 ± 12%, *p* < 0.0001). Exposure to the highest drug concentration (125 µM) also resulted in a significant increase in surrogate stroke volume (SSV: +75 ± 48%, *p* = 0.003), whereas nonsignificant increases were observed for dorsal aorta diameter (+11 ± 9%) and flow (+26 ± 28%). The atrium–ventriculum (A–V) beat ratio was not affected at any concentration. In contrast to the 1-h exposure, exposure for 48 h reversed the direction of the effect on dorsal aorta diameter and flow, resulting in a dose-dependent decrease in dorsal aorta flow, which reached −44 ± 20% of control values (*p* = 0.023) at the highest concentration (16 µM) (**Figure 2**). Exposure to the same concentration resulted in a nonsignificant reduction in dorsal aorta diameter (−9 ± 7%). Significant reductions of both atrial and ventricular beat rate (*p* < 0.001) were observed at all exposure concentrations (2, 4, 8, and 16 µM). The magnitude of the decrease was approximately −40 ± 13% of control values at the highest exposure concentration.

#### Losartan

Exposure to losartan for 1 h did not result in any statistically significant effects on any of the measured endpoints (**Figures 2** and **3**). The highest effect size was observed for dorsal aorta flow (+32% ± 34%) and surrogate stroke volume (+30% ± 36%) after exposure to 5 mM, but within-group variability meant that these effects did not achieve statistical significance. Conversely, exposure to losartan for 48 h resulted in the significant reduction of both atrial and ventricular beat rate in zebrafish exposed to the highest concentration (5 mM) (ABR: −17 ± 5%, *p* = 0.0006; AVR: −17 ± 5%, *p* = 0.0012). Surrogate stroke volume was also increased at the lowest and highest concentration only by 34 ± 21% and 30 ± 33%, respectively, although this effect was not statistically significant.

#### Captopril

Exposure to captopril for 1 h (**Figures 2** and **3**) resulted in a significant decrease in dorsal aorta blood flow and surrogate stroke volume at 6.25 mM (DA blood flow: −43 ± 13%; *p* = 0.0350; SSV: −39 ± 15%, *p* = 0.0213). The decrease in both parameters was also observed at higher exposure concentrations, although in this case, the effect was not statistically significant. A nonsignificant increase in dorsal aorta diameter (+10 ± 9%) was also observed in fish exposed to the highest drug concentration (50 mM). No effects were observed, however, for atrial and ventricular beat rate or A–V beat ratio. Exposure to captopril for 48 h only resulted in nonsignificant dose-dependent trends: an increase in DA diameter, which reached an effect size of +13 ± 18% after exposure to 50 mM and a decrease in DA flow, which reached an effect size of −31 ± 29% after exposure to 25 mM.

#### Time-Dependent Changes of Zebrafish Cardiovascular Parameters in the Control Group

In 3 out of 15 cases, the values of cardiovascular parameters in control zebrafish were significantly different in the 1- and 48-h exposure experiments. Specifically, DA blood flow and SSV in the losartan-treated group (i.e., higher values observed at 48 h; *p* < 0.05) and DA diameter in the captopril-treated group (i.e., lower values observed at 48 h; *p* < 0.05). It is important to note that the 1- and 48-h exposures to the test compounds were carried out as independent experiments. These statistical differences are lost once the experiment-specific control values are compared to the historical control data pooled from the six different experiments described here. This suggests that the observed differences fall within the normal interexperiment variability observed in zebrafish laboratories and, in this case, is unlikely to affect the interpretation of drug-mediated effects.

### Meta-Analysis of Effect Size and Direction in Zebrafish, Rat, Dog, and Human

**Figure 4:**
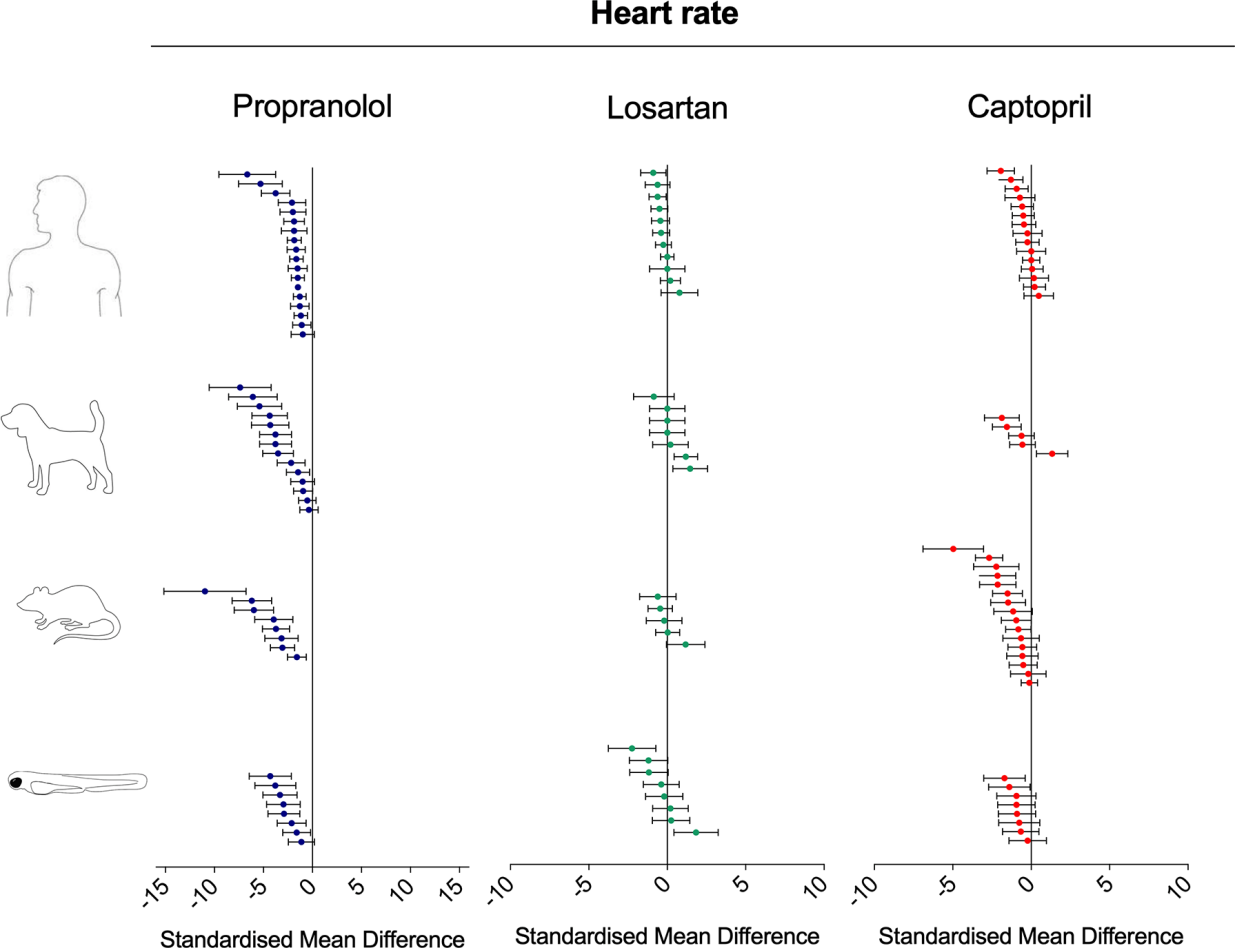
Overview of the effects of propranolol, losartan, and captopril on the heart rate of human, dog, rat, and zebrafish. Data are expressed as standardized mean difference (treated vs. control) ± 95% confidence interval. The data related to human, dog, and rat were retrieved from the literature, whereas the zebrafish data were generated in the present study. Each data point represents a different treatment group. The same dataset was used to perform a quantitative meta-analysis. A detailed description of the results is provided in the **Supplementary Material Data Sheet 1**.

**Figure 5:**
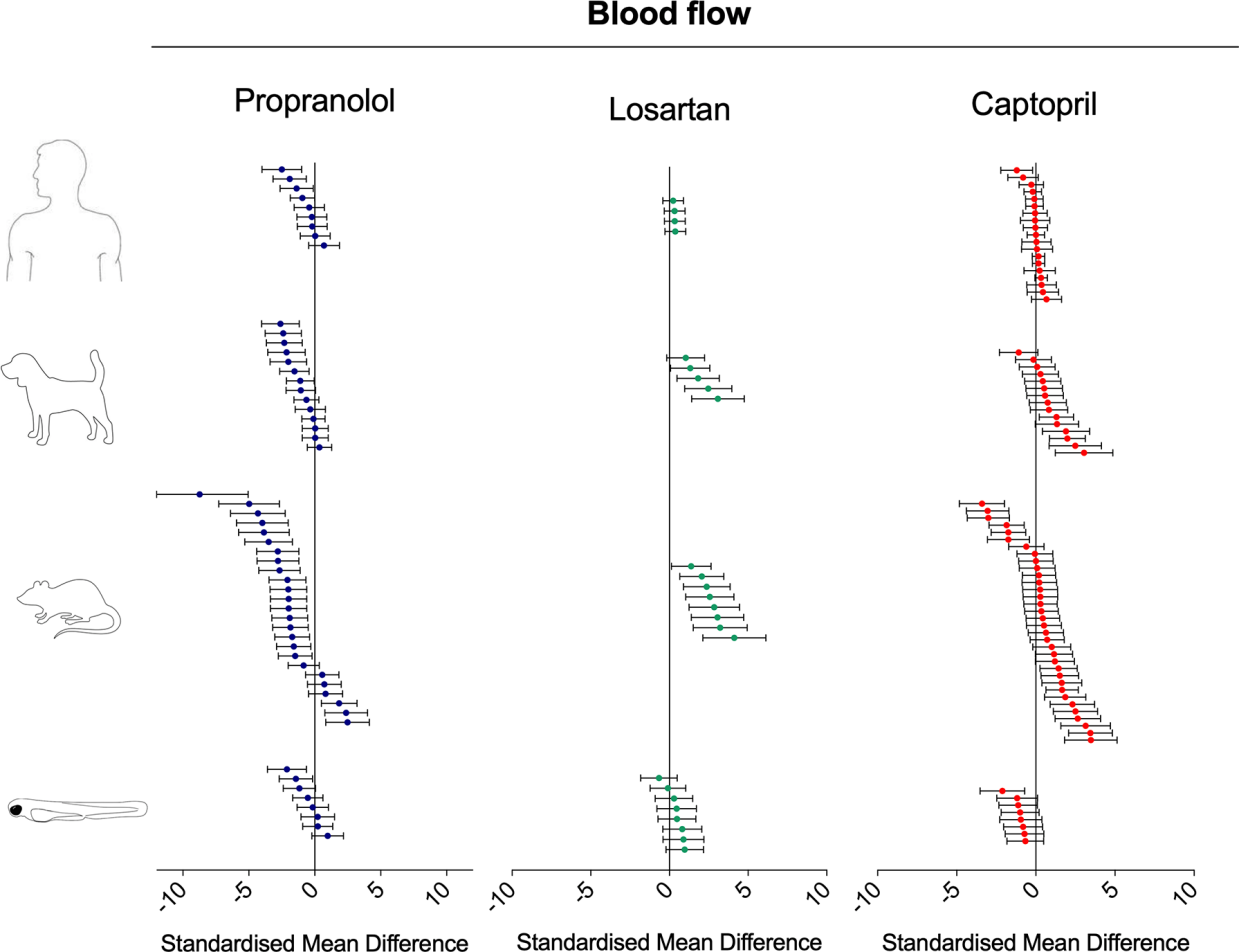
Overview of the effects of propranolol, losartan, and captopril on the blood flow of human, dog, rat, and zebrafish. Data are expressed as the standardized mean difference (treated vs. control) ± 95% confidence interval. The data from human, dog, and rat were retrieved from the literature, whereas the zebrafish data were generated in the present study. Each data point represents a different treatment group. The same dataset was used to perform a quantitative meta-analysis. A detailed description of the results is provided in the **Supplementary Material Data Sheet 1**.

**Figure 6:**
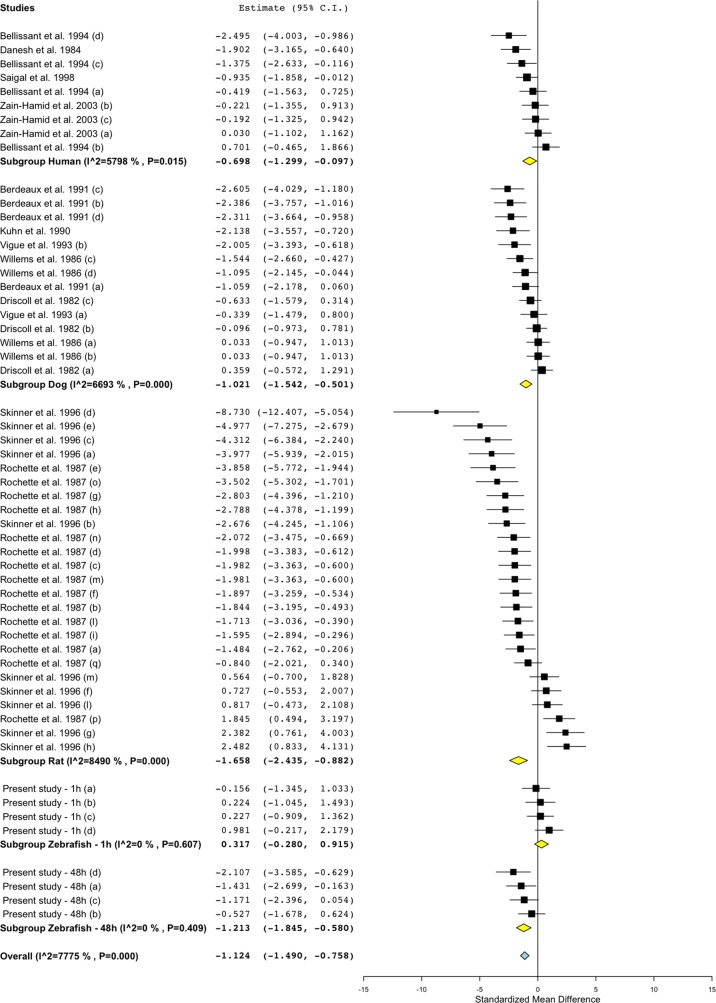
Meta-analysis of the effects of propranolol on blood flow in zebrafish, rat, dog, and humans. Effect size reported as standardized mean difference ± 95% confidence interval.

**Figure 7:**
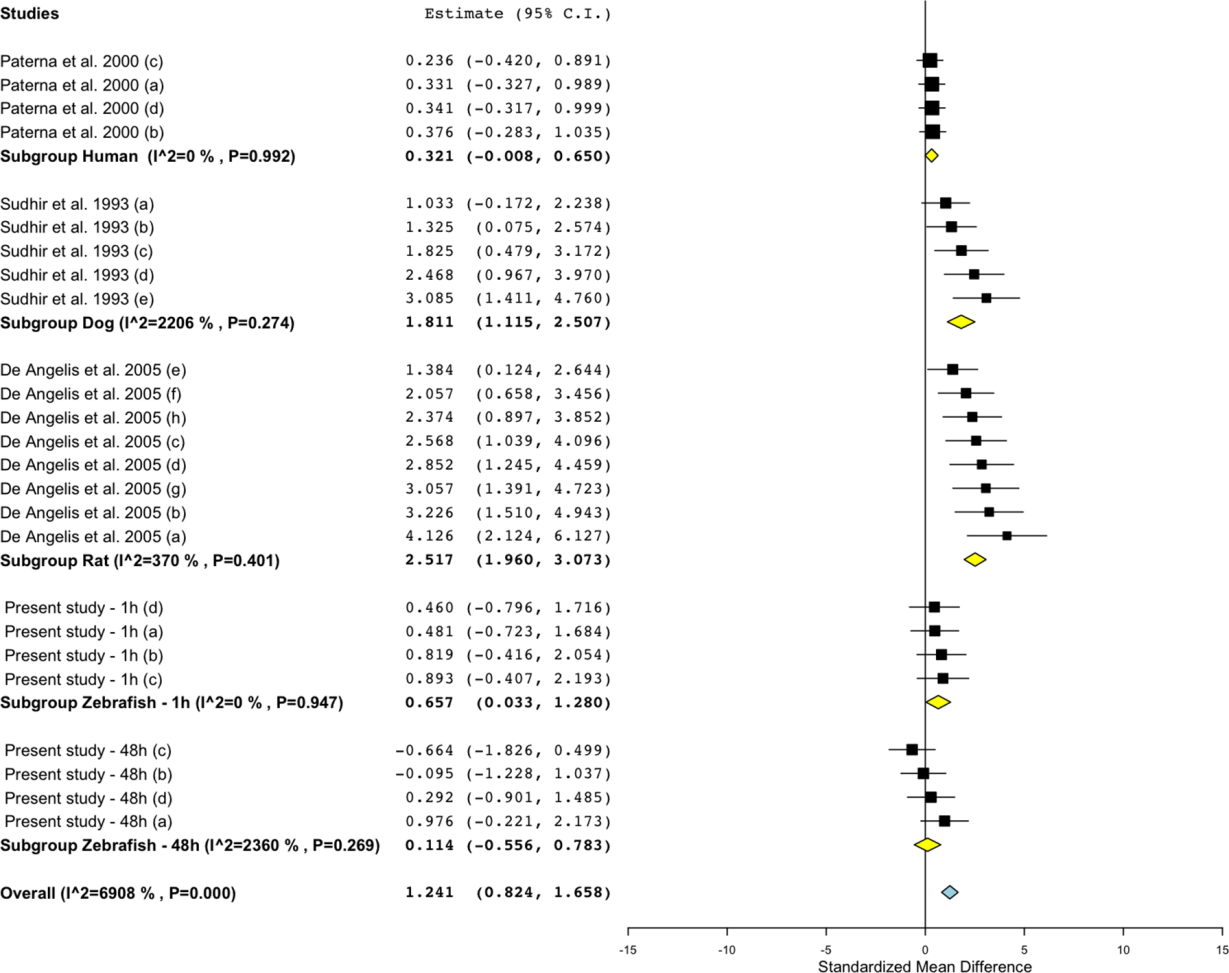
Meta-analysis of the effects of losartan on blood flow in zebrafish, rat, dog, and humans. Effect size reported as standardized mean difference ± 95% confidence interval.

**Figure 8:**
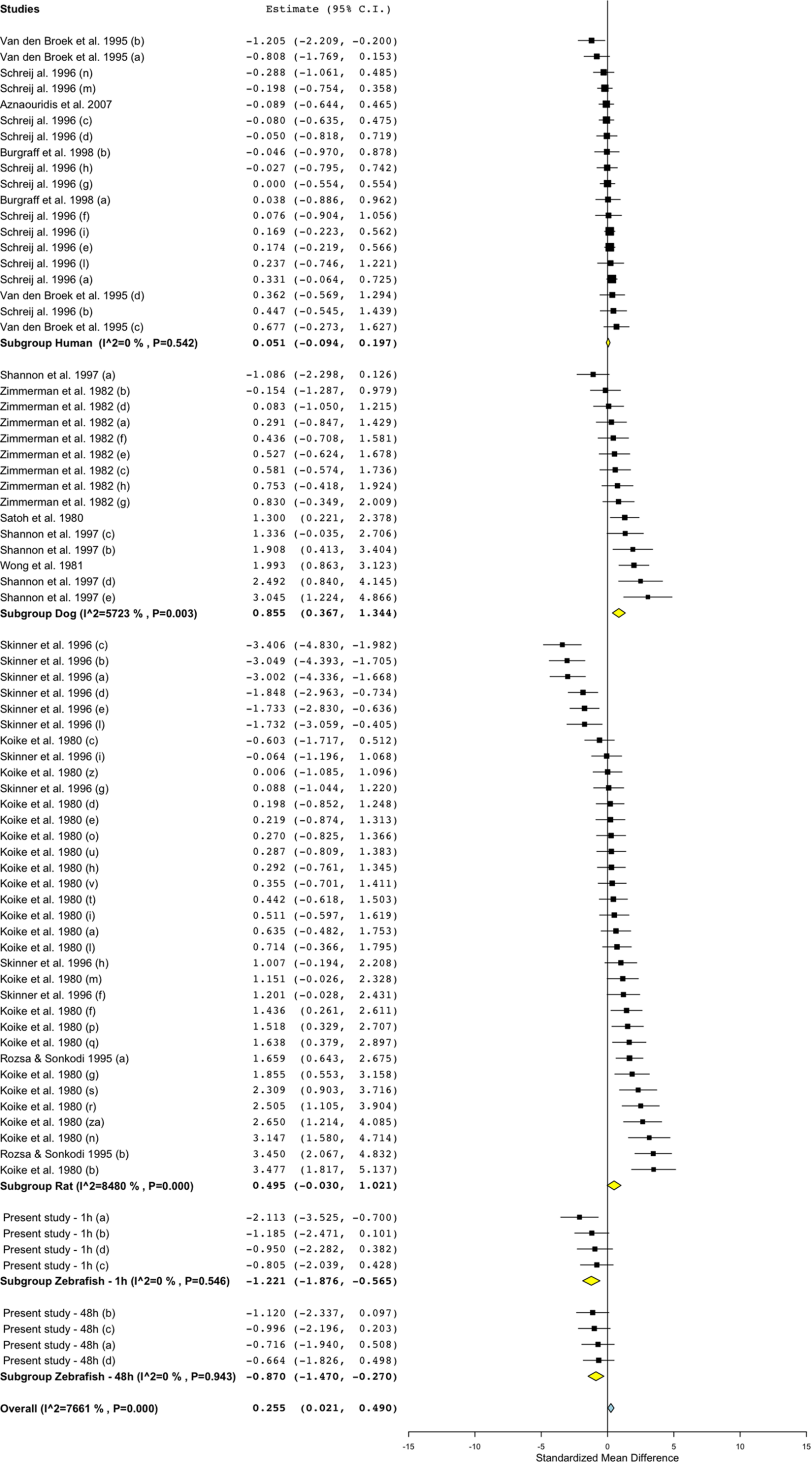
Meta-analysis of the effects of captopril on blood flow in zebrafish, rat, dog, and humans. Effect size reported as standardized mean difference ± 95% confidence interval.

**Figure 9:**
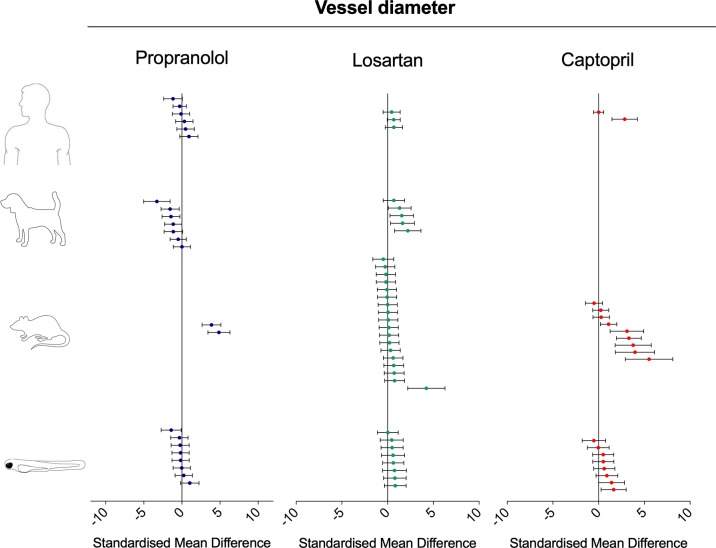
Overview of the effects of propranolol, losartan, and captopril on the blood vessel diameter of human, dog, rat, and zebrafish. Data are expressed as the standardized mean difference (treated vs. control) ± 95% confidence interval. The data related to human, dog, and rats were retrieved from the literature, whereas the zebrafish data were generated in the present study. Each data point represents a different treatment group. The same dataset was used to perform a quantitative meta-analysis. A detailed description of the results is provided in the **Supplementary Material Data Sheet 1**.

**Figure 10:**
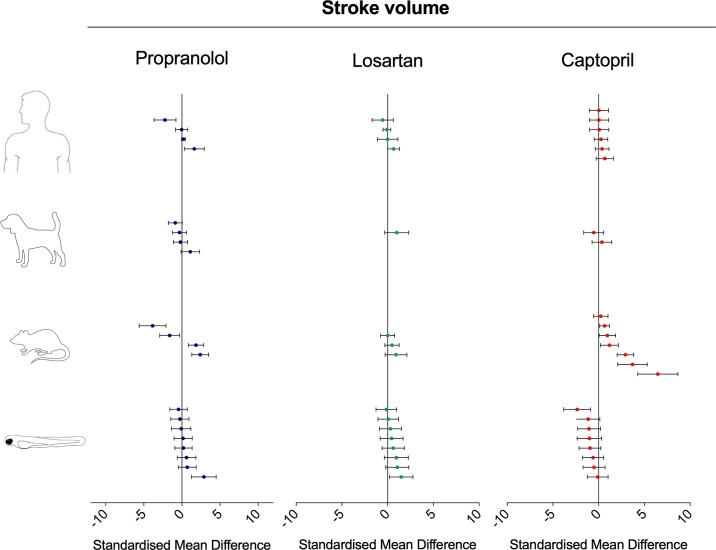
Overview of the effects of propranolol, losartan, and captopril on the stroke volume of human, dog, rat, and zebrafish. Data are expressed as the standardized mean difference (treated vs. control) ± 95% confidence interval. The data related to human, dog, and rat were retrieved from the literature, whereas the zebrafish data were generated in the present study. Each data point represents a different treatment group. The same dataset was used to perform a quantitative meta-analysis. A detailed description of the results is provided in the **Supplementary Material Data Sheet 1**.

To investigate the relevance of zebrafish cardiovascular responses to those observed in two preclinical mammalian species (rat, dog), as well as in humans, we performed a quantitative meta-analysis across four endpoints: heart rate, blood flow, surrogate stroke volume/stroke volume, and blood vessel diameter. We identified a total of 23 suitable studies for propranolol (**Table 1**), 18 for losartan (**Table 2**), and 31 for captopril (**Table 3**). Beyond the data generated here, the only relevant zebrafish data identified in the literature referred to the effects of propranolol on zebrafish heart rate (i.e., three studies). The database of the extracted data and the characteristics of each study are provided in the **Supplementary Material Table 1**. As expected, during data extraction, we observed a wide diversity of experimental conditions used across studies including different doses, administration routes (intravenous, oral), underlying health status of the experimental subjects (e.g., healthy vs. disease models), data collection procedures (e.g., invasive vs. noninvasive), and duration of the treatment (e.g., from bolus dosage to sustained administration over several months). This diversity resulted in a significant heterogeneity of the dataset; nonetheless, our objective was specifically to establish whether drug-induced effects in zebrafish were comparable in terms of effect size and direction to the ones observed in other species, rather than performing an accurate analysis of the efficacy of the drug. For this reason, to minimize potential artifacts deriving from subselection of specific conditions, we included all available data in the analysis. **Figures 4**, **5**, **9**, and **10** display a simplified standardized mean difference for each drug and for each species. **Figures 6**, **7**, and **8** display the detailed meta-analysis of drug effects on blood flow, one of the two endpoints (together with stroke volume) for which we observed a divergence of zebrafish response to captopril from that observed in mammals. The detailed meta-analysis of all the other drug-endpoint combinations is provided in the **Supplementary Material Data Sheet 1** (**Supplementary Figures 1–9**).

**Table 1:** List of studies involving the treatment of different species with propranolol.

Species	Study	Endpoint*	Control sample size	Treatment sample size	Health status of the experimental subjects
Human	Bellissant et al., 1994	HR, BF, VD, SV	6–7	6–7	Healthy
Human	Crawford et al., 1980	HR	10–19	10–19	Healthy
Human	Danesh et al., 1984	BF	7	7	Disease
Human	Le Winter et al., 1975	HR	10	10	Healthy
Human	Marshall et al., 1981	HR	7	18	Healthy/disease
Human	Mo et al., 2011	HR	126	126	Healthy/disease
Human	Morris et al., 1983	HR	22	22	Disease
Human	Port et al., 1980	HR, SV	12	12	Healthy
Human	Saigal et al., 1998	HR, BF, VD	10	10	Disease
Human	Zain-Hamid et al., 2003	HR, BF, VD	12	4	Disease
Dog	Berdeaux et al., 1991	HR, BF, VD	7	7	Healthy
Dog	Driscoll et al., 1982	HR, BF, SV	9–10	9–10	Healthy
Dog	Kuhn et al., 1990	BF, VD, SV	6	6	Healthy
Dog	Vigue et al., 1993	HR, BF, VD	6	6	Healthy
Dog	Willems et al., 1986	HR, BF	8	8	Disease
Rat	Chillon and Baumbach, 1999	VD	16	13	Disease
Rat	Gay et al., 1990	HR, SV	12	10	Healthy/Disease
Rat	Hatzinikolaou et al., 1983	HR, SV	7	7	Healthy
Rat	Rochette et al., 1987	HR, BF, SV	6	6	Healthy
Rat	Skinner et al., 1996	HR, BF	5–9	7–9	Healthy/disease
Zebrafish	Finn et al., 2012	HR	20	20	Healthy
Zebrafish	Fraysse et al., 2006	HR	48	48	Healthy
Zebrafish	Schwerte et al., 2006	HR	7	7	Healthy
Zebrafish	Present study	HR, BF, VD, SV	4–6	4–6	Healthy

*HR, ^#^Heart rate; BF, Blood flow; VD, Vessel diameter; SV, Stroke volume.

**Table 2:** List of studies involving the treatment of different species with losartan.

Species	Study	Endpoint*	Control sample size	Treatment sample size	Health status of the experimental subjects
Human	Craft, 1997	HR, SV	6	6	Disease
Human	Crozier et al., 1995	HR	26	22–29	Disease
Human	Hartog et al., 2016	HR, SV	19–42	19–42	Disease
Human	Gismondi et al., 2015	VD	16	16	Disease
Human	Kekovic´ et al., 2012	HR	30	30	Disease
Human	Konstam et al., 2000	HR	13	13	Disease
Human	Schiffrin et al., 2000	VD	9	9	Disease
Human	Paterna et al., 2000	BF	18	18	Disease
Dog	Lambert, 1995	HR	6	6	Healthy
Dog	Lynch et al., 1999	HR	8	8	Disease
Dog	MacFadyen et al., 1992	HR	16	16	Disease
Dog	Sudhir et al., 1993	HR, BF, VD	6	6	Healthy
Dog	Suzuki et al., 2001	HR, SV	5	5	Disease
Rat	Azevedo et al., 2003	HR, SV	16	11	Disease
Rat	De Angelis et al., 2005	HR, BF, SV	6	6	Healthy
Rat	Koprdova et al., 2009	VD	6	6	Healthy/disease
Rat	Matrougui et al., 2000	VD	7	7	Healthy/disease
Rat	Song et al., 2015	HR	6	6	Healthy
Zebrafish	Present study	HR, BF, VD, SV	4–6	4–6	Healthy

*HR, Heart rate; BF, Blood flow; VD, Vessel diameter; SV, Stroke volume.

**Table 3:** List of studies involving the treatment of different species with captopril.

Species	Study	Endpoint*	Control sample size	Treatment sample size	Health status of the experimental subjects
Human	Aznaouridis et al., 2007	VD	25	25	Disease
Human	Burggraaf et al., 1998	HR, BF	9	9	Healthy
Human	Chau et al., 1992	VD	8	8	Disease
Human	Cleland et al., 1991	HR	16	16	Disease
Human	Leier et al., 1983	SV	7	7	Disease
Human	Massie et al., 1982	HR, SV	14	14	Disease
Human	Sturani et al., 1982	HR	15	15	Disease
Human	Vandenburg et al., 1983	HR	9	9	Disease
Human	Konstam et al., 2000	HR	16	16	Disease
Human	Schreij et al., 1996	BF	8–50	8–50	Disease
Human	Van den Broek et al., 1995	BF	9	9	Disease
Human	Schanzenbacher and Liebau, 1983	SV	9	9	Disease
Dog	Blackford et al., 1990	SV	10	5	Disease
Dog	Jugdutt, 1995	HR	12	12	Disease
Dog	Lynch et al., 1999	HR	9	10	Disease
Dog	Satoh et al., 1980	BF	8	8	Healthy
Dog	Shannon et al., 1997	HR, BF	5–9	5–9	Disease
Dog	Wong et al., 1981	BF	9	9	Disease
Dog	Zimmerman et al., 1982	BF	6	6	Disease
Rat	Silva et al., 2002	HR	10	7–10	Healthy
Rat	Freslon and Giudicelli, 1983	VD	10	10	Disease
Rat	Jin et al., 2001	HR	6	6	Disease
Rat	Kimura et al., 1991	VD	5–6	5	Disease
Rat	Koike et al., 1980	BF	7	6–7	Disease
Rat	Miguel-Carrasco et al., 2010	HR	6	6	Healthy
Rat	Pfeffer et al., 1982	SV	11–13	9	Healthy/disease
Rat	Pfeffer et al., 1985	SV	8–36	8–23	Healthy/disease
Rat	Raya et al., 1989	HR	9	7	Disease
Rat	Rozsa and Sonkodi, 1995	BF	10	10	Healthy/disease
Rat	Skinner et al., 1996	HR, BF	6–14	6	Healthy/disease
Rat	Wang and Prewitt, 1991	VD	9–11	9–11	Healthy
Zebrafish	Present study	HR, BF, VD, SV	4–6	4–6	Healthy

*HR, Heart rate; BF, Blood flow; VD, Vessel diameter; SV, Stroke volume.

#### Interspecies Drug-Induced Effects on Heart Rate

Treatment with the beta-adrenergic receptor antagonist propranolol was associated with a significant decrease of heart rate in all species (**Figure 4**). The effects observed in zebrafish were comparable with those in other species, both in terms of effect direction (dose–response decrease) and effect size (standardized mean difference (SMD): zebrafish-1h = −1.84; zebrafish-48h = −3.46; zebrafish-other studies = −1.79; rat = −4.31; dog = −2.92; human = −1.71). Conversely, the effects of the angiotensin II receptor antagonist losartan were less clear (**Figure 4**), with observed SMDs oscillating between negative and positive values in all species, except for zebrafish treated for 48 h (SMD: zebrafish-1h = +0.45; zebrafish-48h = −1.14; rat = −0.07; dog = +0.36; human = −0.30). For all species, whereas some studies reported a decrease in heart rate, others reported an increase. Despite these discrepancies, overall zebrafish responses after 1 h of exposure were broadly comparable with the effect range observed in mammals. The effects on heart rate were more consistent when the renin−angiotensin system was modulated at the level of the angiotensin-converting enzyme by the ACE inhibitor captopril (**Figure 4**). Treatment with this compound was associated with an overall decrease in heart rate in all species, with only two exceptions, represented by one study in humans (Burgraaff et al., 1998) and one in dogs (Lynch et al., 1999) (SMD: zebrafish-1h = −0.64; zebrafish-48h = −1.21; rat = −1.29; dog = −0.65; human = −0.39). Furthermore, in this case, zebrafish responses were directly comparable with those observed in mammals.

#### Interspecies Drug-Induced Effects on Blood Flow

Treatment with propranolol, in the vast majority of cases, was associated with a significant decrease in blood flow in all species (**Figure 5**; **Figure 6**; SMD: zebrafish-1h = +0.32; zebrafish-48h = −1.21; rat = −1.66; dog = −1.02; human = −0.70), although a small number of studies across all species reported an increase in blood flow in some of the experimental groups (human: Bellissant et al., 1994; dog: Driscoll et al., 1982; rat: Rochette et al., 1987; Skinner et al., 1996; zebrafish: present study—zebrafish-1h). In those cases, the highest SMD was +0.98 for zebrafish-1h, +2.48 for rat, +0.36 for dog, and +0.70 for human.

Conversely, the meta-analysis revealed that pharmacological modulation of the renin–angiotensin system by losartan was associated with an overall positive effect on blood flow in all species (**Figures 5** and **7**). SMD values for losartan were as follows: zebrafish-1h = +0.66; zebrafish-48h = +0.11; rat = +2.52; dog = +1.81; human = +0.32. However, only one study, including multiple data points, was identified for each species for this specific endpoint; thus, these findings should be treated with caution (human: Paterna et al., 2000; dog: Sudhir et al. 1993; rat: De Angelis et al., 2005).

Differently from the responses to propranolol and losartan, zebrafish responses to the ACE inhibitor captopril revealed an overall inconsistency between zebrafish and mammalian changes in blood flow. In fact, captopril induced a consistent decrease in blood flow in zebrafish after both 1- and 48-h exposure, whereas the overall effect was positive in rat and dog and close to zero in humans (**Figure 5**, **Figure 8**; SMD: zebrafish-1h = −1.22; zebrafish-48h = −0.87; rat = +0.50; dog = +0.86; human = +0.05). Interestingly, one study using rat (Skinner et al., 1996) and one in dog (Shannon et al. 1997) also reported a significant decrease in blood flow after treatment with captopril (lowest SMD, −3.40 and −1.09, respectively).

#### Interspecies Drug-Induced Effects on Blood Vessel Diameter

Both adrenergic- and angiotensin-mediated mechanisms are known to be involved in the regulation of vasocontraction and vasodilation. Although the pharmacological modulation of the renin–angiotensin system *via* losartan and captopril was associated with the predicted effect (i.e., vasodilation), beta-adrenergic modulation *via* propranolol treatment produced conflicting results both within, and between species (**Figure 9**). The vasodilation induced by losartan was observed in all species, with striking similarities between zebrafish, dog, and humans, both in terms of effect direction, and magnitude (SMD: zebrafish-1h = +0.58; zebrafish-48h = +0.54; rat = +0.22; dog = +1.41; human = +0.61). The vasodilation induced by captopril was more obvious in rat than in other species, although no data were available from dog studies (SMD: zebrafish-1h = +0.31; zebrafish-48h = +0.87; rat = +2.03; human = +1.36). Only two human studies were identified, of which one showed no effects (SMD = +0.007) and one significant vasodilation (SMD = +2.86). The effect induced by propranolol on blood vessel diameter regulation was not as clear as that observed for both losartan and captopril. The observed SMDs for propranolol were as follows: zebrafish-1h = +0.166; zebrafish-48h = −0.37; rat = +4.30; dog = −1.16; human = +0.02. At an intraspecies level, both in zebrafish and human, some treatments induced vasodilation, whereas others resulted in vasoconstriction (min–max CI: zebrafish-1h = −0.33/+1.04; human: −1.17/+0.92). In the case of the zebrafish, vasoconstriction was observed at the lowest exposure concentration, whereas vasodilation occurred at the higher levels. At an interspecies level, studies performed in rat and dog showed diametrically opposite effects, with significant vasodilation in rat (SMD = +4.30) and consistent vasoconstriction in dog (SMD = −1.16).

#### Interspecies Drug-Induced Effects on (Surrogate) Stroke Volume

Treatment with propranolol induced contrasting intraspecies changes in stroke volume (in the case of zebrafish, measured as surrogate stroke volume) (**Figure 10**; SMD: zebrafish-1h = +0.89; zebrafish-48h = −0.04; rat = −0.23; dog = −0.14; human = −0.03). All species displayed both increased and decreased values for the same endpoint (min–max SMD: zebrafish-1h = −0.26/+2.88; zebrafish-48h = −0.50/+0.21; rat = −3.85/+2.40; dog = −0.87/+1.11; human = −2.21/+1.63). Losartan treatment was associated with an overall increase in stroke volume in all species (**Figure 10**; SMD: zebrafish-1h = +0.58; zebrafish-48h = +0.57; rat = +0.36; dog = +0.99; human = +0.084). A similarity was observed between zebrafish and mammalian responses, both in terms of effect direction and size. Conversely, the zebrafish response to captopril tended to be in contrast with the mammalian responses, particularly with those measured in rat and human (**Figure 10**; SMD: zebrafish-1h = −1.33; zebrafish-48h = −0.52; rat = +2.03; dog = −0.08; human = +0.28).

## Discussion

Here, we provide evidence that zebrafish cardiovascular responses to propranolol, losartan, and captopril are largely in agreement with those observed in humans, both in terms of effect size and direction, revealing a striking similarity between the two species. Specifically, zebrafish responses recapitulated those observed in humans, in terms of both effect size and direction, in over 80% of the cases we assessed. However, in some of these cases, the evaluation of the similarity of the effect direction is not univocal, as the same drug caused contrasting effect directions within the same species. In those cases, our comparability assessment was based on the range of observed effects rather than on the standardized mean effect direction.

Beta-adrenergic receptors are key regulators of cardiovascular homeostasis. Beta-blockers, such as propranolol, cause a competitive inhibition of the beta-adrenergic receptors, countering the effects of catecholamines (Ladage et al., 2013). The clinically relevant outcomes of such inhibition include the reduction of heart rate and force of cardiac muscle contraction. In teleost fish, the beta-adrenergic system mediates a diverse range of functions as it does in humans, including the modulation of cardiac output (Altimiras et al., 1995), cardio-ventilatory responses (McKenzie et al., 1995), metabolic regulation (Van Heeswijk et al., 2006), and skeletal muscle performance (McDonald et al., 1989). In 2007, Owen et al. reviewed the comparative pharmacology of beta-adrenergic receptor antagonists in fish and humans, highlighting not only the apparent high degree of functional and evolutionary conservation of the beta-adrenergic system but also the need to advance the understanding of beta-adrenergic-mediated functions in fish species (Owen et al., 2007). Recorded observations of beta-blockers-induced cardiovascular effects in fish date back to the 1960s (Randall and Stevens, 1967). In the 1970s, Payan and Girard (1977) used perfusion techniques and exposure to two adrenergic blockers (phentolamine and propranolol) to dissect the individual contribution of alpha and beta-adrenergic responses to the vasodilatory effects induced by epinephrine in the trout. Subsequent experiments with zebrafish larvae have mainly been focused on the heart, demonstrating that propranolol decreases heart rate (Fraysse et al., 2006; Schwerte et al., 2006; Finn et al., 2012) without alteration of QT interval (Milan et al., 2006). Our data not only confirmed previous observations but also shed new light on the time-dependent effects of the drug on an a set of other important cardiovascular parameters—such as blood flow, atrium–ventriculum beat ratio, aorta diameter, and stroke volume—providing an integrated profile of the drug-mediated cardiovascular effects that would be difficult to obtain using mammalian preclinical species (Parker et al., 2014).

It is important to note that the effects observed in zebrafish after 1 h exposure to propranolol were sometimes different from those observed after 48 h exposure. For example, whereas the inhibitory effect of propranolol on heart rate was consistent at both time points, the effect on dorsal aorta blood flow shifted from positive to negative, and the increased surrogate stroke volume observed at 1 h returned to control values after 48 h of exposure. These differences are likely driven by a combination of pharmacokinetic (PK) and pharmacodynamic (PD) processes (Vauquelin and Charlton, 2010). Human and preclinical mammalian studies are generally performed by administering a single dose or repeated doses of drug at regular intervals orally or *via* injection. Conversely, zebrafish experiments are mainly carried out using immersion exposure in which the animals remain in contact with the drug continuously until the end of the experiment. The different administration strategies adopted in different experiments is likely to produce different PK/PD profiles both within one species and among different species, which may act as confounding factor and affect the translational value of the experiments. If the tested drug is chemically stable in water, waterborne exposure is likely to produce sustained (rather than oscillatory) internal drug concentrations in the zebrafish over time. In turn, this may generate exposure-specific drug/target interaction dynamics that can ultimately result in variable time-dependent phenotypic effects (Margiotta-Casaluci et al. 2016). Beyond experiment-specific PK/PD considerations, it could be hypothesized that the propranolol-mediated elevation of apical functional cardiovascular parameters (i.e., stroke volume and blood flow velocity) in healthy zebrafish may not be sustained for 48 h because of structural/energetic/compensatory limitations (Vatner et al., 2000) despite the sustained blockade of the beta-adrenergic receptor. However, additional time-course experiments would be required to clarify this aspect. Considering the evidence discussed above, we propose that the potential confounding role of exposure dynamics should be explicitly considered as early as possible during the study design phase to maximize the translational value of future zebrafish experiments and avoid data misinterpretation.

Despite the potential differences between internal exposure dynamics in the different species considered in our analysis, the overlap between the range of zebrafish responses and those observed in humans appeared to be significant in terms of both effect size and direction, supporting previous suggestions of functional conservation of the beta-adrenergic receptor. These phenotypic observations are in line with the results obtained by Steele et al. (2011), who used gene knockdown experiments to characterize the role of the three different isoforms of zebrafish beta-adrenergic receptor (β1AR, β2aAR, and β2bAR) on larval cardiac function.

Considering the 10 clinical studies examined in our analysis, the administration frequency of propranolol to patients was as follows: four times per day/five studies; three times per day/one study; two times per day/one study; and single administration/three studies. As drug administration frequency is only one of the many parameters that characterize the design of each study, this simple example serves to highlight the high heterogeneity in experimental conditions encountered during the data extraction phase. However, the meta-analysis of the effect size data and the related data visualization strategy employed in this study allowed us to identify and quantify emerging patterns for each specific cardiovascular response that could not be appreciated by considering only individual studies in isolation. A second advantage of the meta-analysis approach was the possibility to retrospectively identify and evaluate data points falling outside the predicted patterns of response. For example, the administration of propranolol appeared to produce contrasting effects on blood flow within the same species in humans, zebrafish, and rat. In the latter case, a closer evaluation of the data revealed that almost all data points indicating an increase of blood flow were generated by monitoring different areas of the brain of normotensive Wistar–Kyoto rats exposed to propranolol (Skinner et al., 1996). On the other hand, in the same study, all experiments carried out using spontaneously hypertensive rats caused a marked decrease in the same parameter. This example highlights the important role played by the health state of the animal model employed in the experiments and its potential to affect data interpretation and translational value. Considering the 115 data points used in the cross-species analysis of propranolol-induced effects in human, dog, and rat combined, 76 of those data points were generated using healthy subjects, whereas 39 were generated using subjects with altered cardiovascular physiology. The latter group included experiments carried out using patients with *angina pectoris*, myocardial ischemia, hypertension, and liver cirrhosis; rats displaying spontaneous hypertension or with induced myocardial infarction; and dogs with hypertension, hyperdynamic circulation, liver disease, or pretreated with isoproterenol (beta-adrenoreceptor agonist). It is important to consider that the zebrafish used in the present study were healthy animals tested under “normal” physiological conditions. It is plausible that the effect magnitude and sensitivity of some of the endpoints used in our analysis could have been augmented by introducing relevant alterations of the cardiovascular physiology (e.g., tachycardia, hypertension) or using relevant zebrafish cardiac disease models (Asnani and Peterson, 2014; Keßler et al., 2015; Bournele and Beis, 2016).

The choice between healthy and disease models is generally driven by the aim of the specific study (e.g., safety vs. efficacy assessment); however, some target/phenotype associations may be more easily observable in a perturbed system rather than in healthy system. As discussed for the role of exposure dynamics, this factor should also be considered at the early stage of experimental design, as it may influence the statistical power of the experiment as well as the adopted testing strategy. The zebrafish cardiovascular profiling performed in the present study for the two renin–angiotensin system modulators, losartan and captopril, represents a good example of the challenge mentioned above. Losartan is an angiotensin II type 1 receptor antagonist (AT1 receptor) (Siegl et al., 1995), whereas captopril acts by inhibiting the angiotensin-converting enzyme (ACE) (Dzau, 1990). Both AT1 receptor and ACE are two key components of the renin–angiotensin system (RAS), which regulates the homeostatic control of blood pressure, tissue perfusion, and extracellular volume (Atlas, 2007). Pathophysiological deregulation of the RAAS can lead to hypertension; thus, drugs such as losartan and captopril are used to pharmacologically modulate the RAS and, among the various effects, decrease blood pressure (Abraham et al., 2015). Pharmacodynamic responses common to both drugs include reduction of systemic vascular resistance *via* vasodilation, reduction in blood pressure, and increase in cardiac output (Israili, 2000; Abraham et al., 2015).

The statistical power of preclinical studies is a critically important factor driving costs and data interpretation. In the present study, carried out in zebrafish, both captopril and losartan appeared to cause vasodilation; however, none of the responses at any time point were statistically significant using six animals per treatment group. As a term of comparison, 35 and 89% of losartan data points for, respectively, rat and dog were generated using six or less animals. Conversely, these values are 45 and 68% for captopril, confirming the high heterogeneity of the dataset. Despite this uncertainty, when the effect size was compared across different species, we observed that both losartan and captopril induced effect magnitude ranges in zebrafish in line with those observed in rat, dog, and human studies. It is possible to hypothesize that a zebrafish model with induced vasoconstriction would likely facilitate the statistically significant detection of drug-induced vasodilation using a similar, small number of animals.

At the same time, the overall observed pattern of response emerging from the meta-analysis may be partially explained by the structural boundaries that limit the maximum effect size of aorta diameter. For example, in humans, an aortic diameter 50% larger than baseline value is defined as ectasia, which results in aneurysm formation when the ectasia tolerance limits are exceeded (Hager et al., 2002; Erbel and Eggebrecth, 2006). If we also assume this definition is valid for zebrafish, it implies that a nonlethal drug-induced vasodilation is likely to be lower than 50% of control values. This hypothesis is in agreement with the average effect size observed in zebrafish exposed for 48 h to captopril (+16%) and losartan (+6%). As a term of comparison, the average vasodilation observed in mammalian species exposed to losartan was +18% in rat studies, +20% in dog studies, and +10% in human studies. The detection of this type of effect size using standard statistics would require a higher statistical power than the one used in our experiment or alternatively the use of a model with proven extremely low interindividual variability with respect to the endpoint under investigation.

Beyond vessel diameter, the modulation of the RAS system by losartan and captopril exposure also produced consistent interspecies responses for heart rate, blood flow, and stroke volume. The only two cases where the zebrafish data and that from other models differed stemmed from the effect of captopril on blood flow and stroke volume, which displayed a moderate decrease instead of the neutral or positive effect observed in mammals. It is currently unclear whether these discrepancies are biologically meaningful, and additional studies should be carried out in the future to clarify this point. From an evolutionary standpoint, it is known that the RAS system is conserved in fish. Already in 1973, Nishimura and Ogawa, after reviewing the available evidence concerning the conservation of the RAS system in nonmammals, concluded that the components of the RAS system appeared to be evolutionary conserved in fish but raised doubts about the functional conservation of those components, such as their involvement in the sodium retaining processes observed in mammals (Nishimura and Ogawa, 1973). Subsequent studies have confirmed the evolutionary conservation of the RAS components in teleost fish (Fournier et al., 2012), although the functional conservation of those components, to date, is still not fully understood. Several studies investigating the effects of RAS pharmacological modulation in fish models have generated conflicting results, which have led some authors to hypothesize a low conservation of the sartan binding site on the AT1 receptor (Fuentes and Eddy, 1996; Russell et al., 2001). Kitambi et al. (2009) focused on the vasculature of the eye and attempted a morpholino knockdown of the ACE gene in zebrafish; however, the experiment did not induce any obvious effect on eye blood vessel morphology, possibly due to an incomplete inhibition of ACE expression. In the same study, exposure of zebrafish larvae to the ACE inhibitor enalapril maleate induced vasodilation of intraocular blood vessels but not blood vessels in the trunk. On the other hand, many similarities between zebrafish and mammalian RAS-mediated functions also emerge from other studies. Rider et al. (2017) leveraged the advantages provided by transgenic zebrafish lines to demonstrate that mesonephric renin cells respond to RAS-mediated challenges (including salinity challenge and captopril exposure) in a similar manner in both zebrafish and mammals. Kumai et al. (2014) demonstrated that the RAS is involved in Na^+^ homeostasis in zebrafish larvae. Our results were also generated using noninvasive *in vivo* imaging techniques measuring multiple endpoints simultaneously and suggest high similarity between zebrafish and mammalian cardiovascular responses mediated by AT1 receptor antagonism. ACE inhibition generated comparable responses only for the endpoint vasodilation and heart rate but not for stroke volume and blood flow, confirming, to some extent, the elusive nature of ACE functional conservation between teleost fish and mammals.

The comparison of the effect concentrations ranges of the different compounds tested in the present study brought to light an obvious difference between the three drugs. Whereas propranolol exerted cardiovascular effects in zebrafish in the micromolar range, losartan and captopril acted in the millimolar range. This gap is also observable in human *C*_max_ values but to a much smaller extent (i.e., 2- to 10-fold difference) (Schulz et al., 2012). This difference may be due to a combination of PK/PD factors. First, it is possible that the three drugs have different uptake and PK profile in the zebrafish. For example, the low LogKow of captopril (0.27) suggests that zebrafish may not take up this compound from the surrounding water as effectively as propranolol and losartan (LogKow 3.1 and 3.5, respectively). This implies that water test concentrations may not be the most appropriate unit of comparison and that internal concentrations should be used whenever possible to inform comparative evaluations. On the other side, it is plausible that drug-specific pharmacodynamics contributed to the observed difference in water effect concentrations because of the different role played by beta-adrenergic receptors and renin–angiotensin system in the mediation of cardiovascular functions. Finally, in addition to the evolutionary considerations discussed above, it cannot be excluded that the renin–angiotensin system of zebrafish at 7 dpf may not be fully mature from a molecular and functional perspective; however, to our knowledge, no data are currently available to evaluate the plausibility of this hypothesis.

## Conclusions, Limitations, and Future Perspectives

Our meta-analysis revealed some striking similarities between zebrafish and mammalian responses to three common cardiovascular drugs: propranolol, losartan, and captopril. Our data suggest that, albeit based on data from a limited number of drugs, the cardiovascular effects of both beta-adrenergic receptor and angiotensin II type 1 receptor antagonism can be reliably demonstrated in larval zebrafish. In contrast, treatment to induce ACE inhibition led to results that were only partially in agreement with the known mammalian responses. This uncertainty would suggest that this specific mechanism of action should be considered outside the domain of applicability of the zebrafish model for drug testing, until more robust evidence becomes available.

As already demonstrated previously by Parker et al. (2014), the *in vivo* imaging of zebrafish larvae appears to be a highly valuable approach that enables the noninvasive detection of drug-induced integrated cardiovascular effects. Nonetheless, it is important to highlight certain shortcomings of our study that may have affected, in some cases, the degree of reliability of the overall results. The first and most obvious limitation is the relatively low statistical power of the experiments, carried out using six animals per treatment group. The selection of this design was driven both by previous experiments (Parker et al., 2014), by the practical aspects of the *in vivo* imaging process, and by the aim of generating a procedure suitable for higher-throughput testing. Of note, however, is that this limitation applies not only to our study but also to several papers from which we extracted the mammalian data used in our meta-analysis. As far as zebrafish tests are concerned, the data we generated can be used to set the statistical power of future experiments and achieve an optimal design (e.g., by increasing sample size in line with the objective of the experiment). In some cases, the sensitivity of the experiment could be increased by testing a cardiovascular disease model or by employing genetically engineered zebrafish strains that express fluorescent tags in specific cells. The latter approach may offer a powerful multiscale perspective on drug action and facilitate the interpretation of apical phenotypic processes.

A second important limitation of our zebrafish test was the lack of data concerning the internal concentration of the drug in the animal. This missing piece of information prevents the full translation of PK/PD dynamics observed in zebrafish to other species. The routine quantification of drug internal concentrations in zebrafish larvae remains technically challenging (e.g., it is difficult to separate the larvae from the exposure medium while minimizing the risk of contaminations or leaching); it requires access to specialized analytical chemistry support and increases the overall cost of each experiment. There are examples where the authors successfully performed such analysis (e.g., Parker et al., 2014), but, in general, those studies remain an exception rather than the rule. Previous studies have demonstrated the importance of internal exposure dynamics to interpret drug-mediated effects in adult fish (Margiotta-Casaluci et al., 2016). It is highly plausible that this aspect is also critically important when larval stages are used. Considering that the routine quantification of drug internal concentrations in zebrafish larvae may remain unrealistic for many laboratories, coordinated efforts aimed at developing PBPK model for the larval life stages may offer a good compromise that would enhance the translational value of the zebrafish model.

The key novel aspect of our work is application of a meta-analysis approach for the quantitative assessment of preclinical model translational potential. This approach, combined with a suitable data visualization strategy, revealed patterns of response that would likely remain undetected by employing more traditional methods of qualitative comparative analysis, including the consideration of a few selected papers as a term of comparison, for example, the use of only statistically significant results (i.e., *p* < 0.05) to guide data interpretation, the employment of textual or table formats to express similarities and differences. The method we used in our study allowed us to zoom out from single studies in an unbiased manner and revealed a surprising overlap of effect magnitudes across species, as well as unexpected intraspecies discrepancies. It also provides a fully transparent platform to evaluate data reproducibility and, in turn, support decision-making. We propose that expanding the meta-analysis of interspecies responses to other target–phenotype combinations in the future will help to precisely define the domain of applicability of zebrafish and increase the confidence in its application. Achieving this goal may help to fully unlock the 3Rs potential of the zebrafish model, which may play a key role in the design of future testing strategies, representing an important and crucial bridge between high throughput *in vitro* and low throughput, high content mammalian *in vivo* testing.

## Data Availability

All datasets generated for this study are included in the manuscript and the supplementary files.

## Ethics Statement

This study was carried out in accordance with the recommendations of the United Kingdom Animals (Scientific Procedures) Act regarding the use of animals in scientific procedures. All the animal studies were carried out at AstraZeneca (United Kingdom) under Project License and Personal Licences granted and approved by the United Kingdom Home Office.

## Author Contributions

MW, LM-C, SO, and MR-W conceived and designed the experiments. MW and LM-C performed the *in vivo* studies. LM-C extracted and analyzed both zebrafish and mammalian data and performed the quality assessment of the dataset. LM-C, MW, SO, and MR-W contributed to the data interpretation. SO and MW contributed with essential materials and equipment. LM-C prepared the figures. LM-C, MW, and SO wrote the manuscript. All the authors reviewed the manuscript.

## Funding

This work was funded by a Biotechnology and Biological Sciences Research Council (BBSRC) Research Grant (BB/100646X/1), cofunded by the AstraZeneca Global Safety, Health and Environment research program, to MRW supporting LMC. The funders were not involved in the study design, collection, analysis, interpretation of data, the writing of this article or the decision to submit it for publications.

## Conflict of Interest Statement

This work was cofunded by the AstraZeneca Global Safety, Health and Environment research program. MW was and SO is an employee of AstraZeneca, a biopharmaceutical company specialized in the discovery, development, manufacturing, and marketing of prescription medicines including propranolol used here. AstraZeneca provided support in the form of salaries for author SO (and for MW during the *in vivo* phase) and cofunded the BBSRC grant to MR-W, which supported LM-C.
