# Supplementary material for: Testing the Translational Power of the Zebrafish: An Interspecies Analysis of Responses to Cardiovascular Drugs
Source: Front Pharmacol. 2019 Aug 16;10:893. doi: 10.3389/fphar.2019.00893 (PMC6707810; doi:10.3389/fphar.2019.00893)
Supplement: Supplementary file 1 [file DataSheet_1.pdf]

Supplementary Material

Supplementary Table 1. Descriptive statistics of seven zebrafish cardiovascular endpoints quantified by in vivo imaging after 1h exposure to propranolol.

Propranolol – Atrium beat rate - 1h					
Treatment (µM)	Ctrl	16	32	64	125
Number of values	5	5	5	6	6
Minimum	210.3	195.7	174.8	171	106.5
25% Percentile	210.5	197.9	177.5	174	125
Median	234	204	189.3	185.7	156.5
75% Percentile	239.2	223	215.9	204.2	171.1
Maximum	241.1	223.4	223.6	217.1	188.3
Mean	226.7	209.2	194.2	189.1	150.7
Std. Deviation	15.01	12.97	20.95	17.08	28.41
Std. Error of Mean	6.713	5.802	10.47	6.974	11.6
Lower 95% CI	208	193.1	160.9	171.1	120.9
Upper 95% CI	245.3	225.3	227.6	207	180.5
**Propranolol – Ventriculum beat rate - 1h**					
Treatment (µM)	Ctrl	16	32	64	125
Number of values	5	5	5	6	6
Minimum	210.2	193.4	174.9	169.1	111.4
25% Percentile	210.5	196.4	177.8	173.4	129.7
Median	237.3	204	189.8	187.3	159.5
75% Percentile	240.8	222.9	214.7	204.2	171.2
Maximum	243.6	223.3	221.9	217.1	189
Mean	228	208.5	194.1	189.3	153.4
Std. Deviation	16.14	13.67	19.99	17.39	26.7
Std. Error of Mean	7.22	6.115	9.997	7.099	10.9
Lower 95% CI	207.9	191.5	162.3	171	125.4
Upper 95% CI	248	225.5	225.9	207.5	181.4
**Propranolol – A:V beat ratio - 1h**					
Treatment (µM)	Ctrl	16	32	64	125
Number of values	5	5	5	6	6
Minimum	0.98	1	0.99	0.98	0.96
25% Percentile	0.9875	1	0.9925	0.995	0.9675
Median	1	1	1	1	0.985
75% Percentile	1	1	1.008	1.005	1
Maximum	1	1	1.01	1.02	1
Mean	0.995	1	1	1	0.9833
Std. Deviation	0.008367	0	0.008165	0.01265	0.01862
Std. Error of Mean	0.003416	0	0.004082	0.005164	0.007601
Lower 95% CI	0.9862	1	0.987	0.9867	0.9638
Upper 95% CI	1.004	1	1.013	1.013	1.003
**Propranolol – Dorsal Aorta Diameter - 1h**					
Treatment (µM)	Ctrl	16	32	64	125
Number of values	6	5	6	6	6
Minimum	11.49	11.74	11.66	12.85	14.01
25% Percentile	12.43	12.33	12.44	13.38	14.14
Median	13.5	12.91	13.25	13.82	14.69
75% Percentile	14.77	13.98	14.09	14.3	15.87
Maximum	15.53	14.51	14.49	14.83	17.41
Mean	13.54	13.1	13.22	13.83	15.05
Std. Deviation	1.421	1.003	0.9867	0.6591	1.261
Std. Error of Mean	0.5801	0.4486	0.4028	0.2691	0.5148
Lower 95% CI	12.05	11.86	12.18	13.14	13.73
Upper 95% CI	15.03	14.35	14.25	14.52	16.38

Propranolol – Average blood flow - 1h					
Treatment (µM)	Ctrl	16	32	64	125
Number of values	6	5	4	6	6
Minimum	0.13	0.09	0.13	0.11	0.16
25% Percentile	0.1375	0.12	0.14	0.1325	0.16
Median	0.165	0.16	0.175	0.16	0.19
75% Percentile	0.1825	0.19	0.1875	0.2125	0.24
Maximum	0.19	0.22	0.19	0.28	0.27
Mean	0.1617	0.156	0.1675	0.1733	0.2
Std. Deviation	0.02317	0.04615	0.0263	0.05888	0.04472
Std. Error of Mean	0.009458	0.02064	0.01315	0.02404	0.01826
Lower 95% CI	0.1374	0.09869	0.1257	0.1115	0.1531
Upper 95% CI	0.186	0.2133	0.2093	0.2351	0.2469
**Propranolol – Linear blood flow - 1h**					
Treatment (µM)	Ctrl	16	32	64	125
Number of values	6	5	4	6	6
Minimum	773.1	554.2	683.5	666.3	857.6
25% Percentile	812.8	714	710.3	797.3	920.5
Median	963	979.9	924.1	905	1034
75% Percentile	1017	1135	1083	1039	1125
Maximum	1086	1189	1091	1110	1172
Mean	934.1	935.4	905.7	907	1024
Std. Deviation	115.6	243.1	200	152.7	114.6
Std. Error of Mean	47.2	108.7	100	62.33	46.77
Lower 95% CI	812.8	633.6	587.5	746.8	904.2
Upper 95% CI	1055	1237	1224	1067	1145
**Propranolol – Surrogate Stroke Volume - 1h**					
Treatment (µM)	Ctrl	16	32	64	125
Number of values	6	5	5	6	6
Minimum	0.03	0.03	0.04	0.04	0.04
25% Percentile	0.0375	0.035	0.045	0.04467	0.0625
Median	0.045	0.05	0.06	0.05	0.09
75% Percentile	0.06	0.055	0.06	0.0675	0.1
Maximum	0.06	0.06	0.06	0.09	0.1
Mean	0.04667	0.046	0.055	0.05604	0.08167
Std. Deviation	0.01211	0.0114	0.01	0.01786	0.02317
Std. Error of Mean	0.004944	0.005099	0.005	0.007293	0.009458
Lower 95% CI	0.03396	0.03184	0.03909	0.03729	0.05736
Upper 95% CI	0.05938	0.06016	0.07091	0.07478	0.106

Supplementary Table 2. Descriptive statistics of seven zebrafish cardiovascular endpoints quantified by in vivo imaging after 48h exposure to propranolol.

Propranolol – Atrium beat rate - 48h					
Treatment (µM)	Ctrl	16	32	64	125
Number of values	6	6	4	6	5
Minimum	223.5	160.6	160.9	158.7	98.6
25% Percentile	229.5	171.3	161.1	159.5	111.1
Median	238.8	182.9	169.3	169.1	142.9
75% Percentile	250.2	213	196.2	192.2	171.8
Maximum	256.7	219.3	202.6	216.7	173.1
Mean	239.5	188.6	175.5	176.2	141.7
Std. Deviation	12	22.32	19.55	21.85	31.65
Std. Error of Mean	4.9	9.112	9.773	8.92	14.15
Lower 95% CI	226.9	165.1	144.4	153.3	102.4
Upper 95% CI	252.1	212	206.6	199.1	181
**Propranolol – Ventriculum beat rate - 48h**					
Treatment (µM)	Ctrl	16	32	64	125
Number of values	6	6	4	6	5
Minimum	223.4	161.2	160.9	154.3	99.1
25% Percentile	229.4	171.3	161.4	158.4	108.1
Median	238.8	182.1	170	164.3	129.9
75% Percentile	250.1	212.8	195.9	172.1	171.7
Maximum	256.8	219.6	202.1	184.9	173
Mean	239.5	188.4	175.7	165.9	137.9
Std. Deviation	12.05	22.29	19.01	10.46	32.75
Std. Error of Mean	4.92	9.1	9.506	4.269	14.65
Lower 95% CI	226.9	165	145.5	154.9	97.24
Upper 95% CI	252.2	211.7	206	176.9	178.6
**Propranolol – A:V beat ratio - 48h**					
Treatment (µM)	Ctrl	16	32	64	125
Number of values	6	6	4	6	5
Minimum	1	1	0.99	0.97	0.99
25% Percentile	1	1	0.9925	0.985	0.995
Median	1	1	1	1	1
75% Percentile	1	1	1	1.125	1.08
Maximum	1	1	1	1.41	1.1
Mean	1	1	0.9975	1.067	1.03
Std. Deviation	0	0	0.005	0.1693	0.04796
Std. Error of Mean	0	0	0.0025	0.06912	0.02145
Lower 95% CI	1	1	0.9895	0.889	0.9705
Upper 95% CI	1	1	1.005	1.244	1.09
**Propranolol – Dorsal Aorta Diameter - 48h**					
Treatment (µM)	Ctrl	16	32	64	125
Number of values	6	6	6	6	5
Minimum	13.1	12.6	12.2	11.6	11.1
25% Percentile	13.1	13.05	12.58	11.98	11.35
Median	13.5	13.6	13.65	13.3	12.4
75% Percentile	14	13.85	14.33	14.85	13.35
Maximum	14.3	14	15	15.3	13.5
Mean	13.57	13.47	13.55	13.38	12.36
Std. Deviation	0.5317	0.5046	1.001	1.432	1.021
Std. Error of Mean	0.2171	0.206	0.4089	0.5845	0.4567
Lower 95% CI	13.01	12.94	12.5	11.88	11.09
Upper 95% CI	14.12	14	14.6	14.89	13.63

Propranolol – Average blood flow - 48h					
Treatment (µM)	Ctrl	16	32	64	125
Number of values	6	6	6	6	5
Minimum	0.2	0.1	0.1	0.1	0.1
25% Percentile	0.2	0.1	0.175	0.1	0.1
Median	0.2	0.15	0.2	0.2	0.1
75% Percentile	0.3	0.2	0.225	0.2	0.15
Maximum	0.3	0.2	0.3	0.2	0.2
Mean	0.2333	0.15	0.2	0.1667	0.12
Std. Deviation	0.05164	0.05477	0.06325	0.05164	0.04472
Std. Error of Mean	0.02108	0.02236	0.02582	0.02108	0.02
Lower 95% CI	0.1791	0.09252	0.1336	0.1125	0.06447
Upper 95% CI	0.2875	0.2075	0.2664	0.2209	0.1755
**Propranolol – Linear blood flow - 48h**					
Treatment (µM)	Ctrl	16	32	64	125
Number of values	6	6	6	6	5
Minimum	983	461.5	951.5	583.2	568.7
25% Percentile	1067	486.9	962.6	743.7	646.8
Median	1172	905.6	1049	955	878.3
75% Percentile	1217	1192	1078	1020	999.9
Maximum	1252	1214	1098	1052	1062
Mean	1147	861.2	1031	891.9	834.3
Std. Deviation	95.52	326.2	59.45	176.9	191.7
Std. Error of Mean	38.99	133.2	24.27	72.22	85.73
Lower 95% CI	1046	518.8	968.5	706.2	596.3
Upper 95% CI	1247	1204	1093	1078	1072
**Propranolol – Surrogate Stroke Volume - 48h**					
Treatment (µM)	Ctrl	16	32	64	125
Number of values	6	6	5	6	4
Minimum	0.04	0.03	0.05	0.04	0.02
25% Percentile	0.04	0.03	0.0525	0.0475	0.025
Median	0.045	0.04	0.06	0.055	0.055
75% Percentile	0.075	0.0625	0.06	0.065	0.07
Maximum	0.09	0.07	0.06	0.08	0.07
Mean	0.055	0.045	0.0575	0.05667	0.05
Std. Deviation	0.02074	0.01761	0.005	0.01366	0.02449
Std. Error of Mean	0.008466	0.007188	0.0025	0.005578	0.01225
Lower 95% CI	0.03324	0.02652	0.04954	0.04233	0.01102
Upper 95% CI	0.07676	0.06348	0.06546	0.071	0.08898

Supplementary Table 3. Descriptive statistics of seven zebrafish cardiovascular endpoints quantified by in vivo imaging after 1h exposure to losartan.

**Losartan – Atrium beat rate - 1h**					
Treatment (mM)	Ctrl	16	32	64	125
Number of values	6	6	5	5	5
Minimum	239.7	226.3	247.3	236	230.7
25% Percentile	241.8	228.3	251.5	236.2	231.8
Median	243.2	251.3	259.8	247.9	235
75% Percentile	247.8	264	265.3	259.3	258.1
Maximum	253.9	267	265.9	268.5	261.8
Mean	244.7	248	258.7	247.7	242.9
Std. Deviation	4.920	17.43	7.53	13.27	14.17
Std. Error of Mean	2.009	7.116	3.368	5.936	6.335
Lower 95% CI	239.5	229.7	249.3	231.3	225.4
Upper 95% CI	249.9	266.3	268	264.2	260.5
**Losartan – Ventriculum beat rate - 1h**					
Treatment (mM)	Ctrl	16	32	64	125
Number of values	6	6	5	5	5
Minimum	239.7	224.9	247.2	236.1	230.5
25% Percentile	241.3	228	251.5	236.2	231.8
Median	242.1	251.3	259.8	250	235.9
75% Percentile	248.4	264.1	265.3	261.9	258.4
Maximum	254.2	267.1	265.9	268.4	261.7
Mean	244.4	247.8	258.7	249.2	243.3
Std. Deviation	5.279	17.81	7.588	13.65	14.15
Std. Error of Mean	2.155	7.272	3.393	6.106	6.326
Lower 95% CI	238.9	229.1	249.2	232.3	225.7
Upper 95% CI	249.9	266.5	268.1	266.2	260.8
**Losartan – A:V beat ratio - 1h**					
Treatment (mM)	Ctrl	16	32	64	125
Number of values	6	6	5	5	5
Minimum	0.980	1	1	0.97	1
25% Percentile	0.995	1	1	0.9925	1
Median	1	1	1	1	1
75% Percentile	1.013	1.003	1	1	1
Maximum	1.020	1.01	1	1	1
Mean	1.002	1.002	1	0.995	1
Std. Deviation	0.004	0.004082	0	0.01225	0
Std. Error of Mean	0.005	0.001667	0	0.005	0
Lower 95% CI	0.9836	0.9974	1	0.9821	1
Upper 95% CI	1.02	1.006	1	1.008	1
**Losartan – Dorsal Aorta Diameter - 1h**					
Treatment (mM)	Ctrl	16	32	64	125
Number of values	5	6	6	5	5
Minimum	11.21	12.96	13.32	12.18	13.4
25% Percentile	11.84	13.08	13.32	12.59	13.48
Median	14.14	13.91	14.62	14.45	14.25
75% Percentile	14.48	14.7	15.11	15.47	14.66
Maximum	14.49	16.52	15.31	15.97	14.81
Mean	13.36	14.09	14.37	14.11	14.1
Std. Deviation	1.46	1.284	0.8671	1.522	0.6111
Std. Error of Mean	0.6531	0.5243	0.354	0.6808	0.2733
Lower 95% CI	11.54	12.74	13.46	12.22	13.35
Upper 95% CI	15.17	15.43	15.28	16	14.86

Losartan – Average blood flow - 1h					
Treatment (mM)	Ctrl	16	32	64	125
Number of values	5	6	6	5	5
Minimum	0.13	0.16	0.17	0.19	0.15
25% Percentile	0.14	0.1675	0.1925	0.19	0.17
Median	0.17	0.175	0.205	0.22	0.19
75% Percentile	0.215	0.2525	0.23	0.370	0.225
Maximum	0.25	0.29	0.26	0.42	0.23
Mean	0.176	0.2017	0.21	0.268	0.196
Std. Deviation	0.04561	0.05193	0.02966	0.1003	0.0313
Std. Error of Mean	0.0204	0.0212	0.01211	0.04488	0.014
Lower 95% CI	0.1194	0.1472	0.1789	0.1434	0.1571
Upper 95% CI	0.2326	0.2562	0.2411	0.3926	0.2349
**Losartan – Linear blood flow - 1h**					
Treatment (mM)	Ctrl	16	32	64	125
Number of values	5	6	6	5	5
Minimum	854.6	870.3	834.2	932	798.1
25% Percentile	866.2	925.7	893.9	956.3	859.1
Median	949	1058	1082	1147	1021
75% Percentile	1028	1280	1220	1260	1177
Maximum	1064	1416	1346	1370	1181
Mean	947.3	1097	1073	1116	1018
Std. Deviation	85.05	205.3	188.7	172.3	164.6
Std. Error of Mean	38.04	83.8	77.03	77.05	73.63
Lower 95% CI	841.7	881.3	874.7	902.2	814
Upper 95% CI	1053	1312	1271	1330	1223
**Losartan – Surrogate Stroke Volume - 1h**					
Treatment (mM)	Ctrl	16	32	64	125
Number of values	5	6	6	5	5
Minimum	0.03	0.04	0.04	0.05	0.04
25% Percentile	0.035	0.04	0.045	0.05	0.045
Median	0.04	0.045	0.05	0.05	0.05
75% Percentile	0.055	0.055	0.055	0.0725	0.05
Maximum	0.06	0.07	0.06	0.08	0.05
Mean	0.044	0.04833	0.05	0.0575	0.048
Std. Deviation	0.0114	0.01169	0.007071	0.015	0.004472
Std. Error of Mean	0.005099	0.004773	0.003162	0.0075	0.002
Lower 95% CI	0.02984	0.03606	0.04122	0.03363	0.04245
Upper 95% CI	0.05816	0.0606	0.05878	0.08137	0.05355

Supplementary Table 4. Descriptive statistics of seven zebrafish cardiovascular endpoints quantified by in vivo imaging after 48h exposure to losartan.

**Losartan – Atrium beat rate - 48h**					
Treatment (mM)	Ctrl	16	32	64	125
Number of values	6	6	6	6	5
Minimum	235.1	219.3	236	209.1	192.1
25% Percentile	239.9	221	237.4	218.6	197.5
Median	243.8	231.1	238.2	229.2	205.5
75% Percentile	264.7	238.9	259.1	236.5	220.7
Maximum	291.8	245.5	260.2	254.8	227
Mean	251.9	230.9	244.9	229.1	208.4
Std. Deviation	20.68	10.15	11.35	14.95	13.08
Std. Error of Mean	8.442	4.143	4.633	6.104	5.852
Lower 95% CI	230.2	220.2	232.9	213.4	192.1
Upper 95% CI	273.6	241.5	256.8	244.8	224.6
**Losartan – Ventriculum beat rate - 48h**					
Treatment (mM)	Ctrl	16	32	64	125
Number of values	6	6	6	6	5
Minimum	234.9	216.1	231.4	209.2	192.1
25% Percentile	239.9	220.2	235.5	218.7	197.6
Median	243.7	231.6	237.9	229.3	205.5
75% Percentile	264.8	248.1	259.1	236.6	220.7
Maximum	292	255.7	260.2	255.2	227
Mean	251.9	233.7	243.8	229.2	208.4
Std. Deviation	20.8	15.13	12.34	15.06	13.07
Std. Error of Mean	8.491	6.176	5.04	6.148	5.847
Lower 95% CI	230.1	217.8	230.9	213.4	192.2
Upper 95% CI	273.7	249.5	256.8	245	224.6
**Losartan – A:V beat ratio - 48h**					
Treatment (mM)	Ctrl	16	32	64	125
Number of values	6	6	6	6	5
Minimum	1	0.92	1	1	1
25% Percentile	1	0.98	1	1	1
Median	1	1	1	1	1
75% Percentile	1	1.003	1.008	1	1
Maximum	1	1.01	1.03	1	1
Mean	1	0.9883	1.005	1	1
Std. Deviation	0	0.03371	0.01225	0	0
Std. Error of Mean	0	0.01376	0.005	0	0
Lower 95% CI	1	0.953	0.9921	1	1
Upper 95% CI	1	1.024	1.018	1	1
**Losartan – Dorsal Aorta Diameter - 48h**					
Treatment (mM)	Ctrl	16	32	64	125
Number of values	6	6	6	6	4
Minimum	10.91	11.34	11.84	11.28	11.96
25% Percentile	11.53	11.57	11.89	11.67	12
Median	12.08	12.2	12.42	13.39	12.76
75% Percentile	12.83	12.57	13.85	14.41	13.74
Maximum	13.22	13.08	15.66	14.5	13.85
Mean	12.12	12.14	12.92	13.12	12.83
Std. Deviation	0.7959	0.6147	1.471	1.325	0.9328
Std. Error of Mean	0.3249	0.251	0.6005	0.5411	0.4664
Lower 95% CI	11.28	11.5	11.37	11.73	11.35
Upper 95% CI	12.96	12.79	14.46	14.51	14.32

Losartan – Average blood flow - 48h					
Treatment (mM)	Ctrl	16	32	64	125
Number of values	6	6	6	6	5
Minimum	0.22	0.26	0.14	0.17	0.21
25% Percentile	0.22	0.275	0.2	0.2	0.225
Median	0.25	0.295	0.275	0.235	0.28
75% Percentile	0.32	0.4	0.3075	0.265	0.335
Maximum	0.32	0.43	0.33	0.28	0.37
Mean	0.2633	0.325	0.2567	0.2317	0.28
Std. Deviation	0.04633	0.06892	0.06772	0.03971	0.06124
Std. Error of Mean	0.01892	0.02814	0.02765	0.01621	0.02739
Lower 95% CI	0.2147	0.2527	0.1856	0.19	0.204
Upper 95% CI	0.312	0.3973	0.3277	0.2733	0.356
**Losartan – Linear blood flow - 48h**					
Treatment (mM)	Ctrl	16	32	64	125
Number of values	6	6	6	6	5
Minimum	1295	1354	1112	1068	1207
25% Percentile	1355	1428	1193	1109	1233
Median	1449	1620	1521	1247	1337
75% Percentile	1551	1731	1613	1314	1436
Maximum	1562	1740	1724	1398	1462
Mean	1446	1586	1446	1228	1335
Std. Deviation	112	153.8	232.5	118.1	104.9
Std. Error of Mean	45.7	62.8	94.9	48.21	46.93
Lower 95% CI	1329	1425	1202	1104	1205
Upper 95% CI	1564	1747	1690	1352	1465
**Losartan – Surrogate Stroke Volume - 48h**					
Treatment (mM)	Ctrl	16	32	64	125
Number of values	6	6	6	6	5
Minimum	0.05	0.07	0.04	0.04	0.06
25% Percentile	0.05	0.07	0.0475	0.0475	0.065
Median	0.06	0.08	0.07	0.06	0.08
75% Percentile	0.0725	0.1	0.0725	0.0725	0.095
Maximum	0.08	0.1	0.08	0.08	0.11
Mean	0.06167	0.08333	0.06333	0.06	0.08
Std. Deviation	0.01169	0.01366	0.01506	0.01414	0.01871
Std. Error of Mean	0.004773	0.005578	0.006146	0.005774	0.008367
Lower 95% CI	0.0494	0.069	0.04753	0.04516	0.05677
Upper 95% CI	0.07394	0.09767	0.07913	0.07484	0.1032

Supplementary Table 5. Descriptive statistics of seven zebrafish cardiovascular endpoints quantified by in vivo imaging after 1h exposure to captopril.

Captopril – Atrium beat rate - 1h					
Treatment (mM)	Ctrl	16	32	64	125
Number of values	6	6	5	5	4
Minimum	219.8	209.6	220	224.8	214.2
25% Percentile	225.1	220.5	224.5	229	214.2
Median	244.7	230.9	229	240.4	236.6
75% Percentile	257.4	240.2	233.7	248.1	237.6
Maximum	261.7	255.8	237.8	249.2	237.6
Mean	242.3	231	229.1	238.9	229.5
Std. Deviation	16.23	15.2	6.301	10.08	13.23
Std. Error of Mean	6.625	6.206	2.818	4.508	7.639
Lower 95% CI	225.3	215.1	221.2	226.4	196.6
Upper 95% CI	259.3	247	236.9	251.4	262.3
**Captopril – Ventriculum beat rate - 1h**					
Treatment (mM)	Ctrl	16	32	64	125
Number of values	6	6	5	5	4
Minimum	239.6	210.1	219	225.5	217.2
25% Percentile	239.7	220.2	224	229	217.2
Median	244.5	230.4	229.3	240.5	238
75% Percentile	257.5	240.2	232.9	252.2	240.5
Maximum	261.9	255.9	235.6	257.3	240.5
Mean	247.7	230.9	228.6	240.6	231.9
Std. Deviation	9.188	15.2	6	12.38	12.79
Std. Error of Mean	3.751	6.205	2.683	5.538	7.385
Lower 95% CI	238	214.9	221.1	225.2	200.1
Upper 95% CI	257.3	246.8	236	255.9	263.7
**Captopril – A:V beat ratio - 1h**					
Treatment (mM)	Ctrl	16	32	64	125
Number of values	6	6	5	5	4
Minimum	0.92	1	0.99	0.97	0.98
25% Percentile	0.9425	1	0.9975	0.9925	0.98
Median	1	1	1	1	0.99
75% Percentile	1	1	1.003	1	1
Maximum	1	1	1.01	1	1
Mean	0.9783	1	1	0.995	0.99
Std. Deviation	0.03488	0	0.006325	0.01225	0.01
Std. Error of Mean	0.01424	0	0.002582	0.005	0.005774
Lower 95% CI	0.9417	1	0.9934	0.9821	0.9652
Upper 95% CI	1.015	1	1.007	1.008	1.015
**Captopril – Dorsal Aorta Diameter - 1h**					
Treatment (mM)	Ctrl	16	32	64	125
Number of values	6	6	5	4	4
Minimum	13.35	13.1	12	11.39	13.79
25% Percentile	13.51	13.34	12.22	11.84	13.9
Median	13.73	13.66	13.89	13.71	15.11
75% Percentile	14.08	16.07	15.21	14.31	16.32
Maximum	14.35	16.29	15.86	14.33	16.42
Mean	13.79	14.35	13.75	13.29	15.11
Std. Deviation	0.3485	1.405	1.576	1.364	1.299
Std. Error of Mean	0.1423	0.5736	0.7047	0.6822	0.6494
Lower 95% CI	13.42	12.88	11.79	11.11	13.04
Upper 95% CI	14.15	15.83	15.7	15.46	17.17

Captopril – Average blood flow - 1h					
Treatment (mM)	Ctrl	16	32	64	125
Number of values	6	6	5	5	4
Minimum	0.16	0.1	0.09	0.08	0.12
25% Percentile	0.1825	0.1	0.11	0.095	0.1275
Median	0.21	0.12	0.14	0.16	0.165
75% Percentile	0.255	0.1559	0.2	0.235	0.21
Maximum	0.3	0.16	0.23	0.26	0.22
Mean	0.2183	0.1257	0.152	0.164	0.1675
Std. Deviation	0.04956	0.0268	0.05215	0.07301	0.04272
Std. Error of Mean	0.02023	0.01094	0.02332	0.03265	0.02136
Lower 95% CI	0.1663	0.09762	0.08724	0.07335	0.09952
Upper 95% CI	0.2703	0.1539	0.2168	0.2546	0.2355
**Captopril – Linear blood flow - 1h**					
Treatment (mM)	Ctrl	16	32	64	125
Number of values	6	6	5	5	4
Minimum	849	563.3	713.5	451.2	737
25% Percentile	868.7	626.9	778.5	586.4	748.4
Median	1070	713.9	880.5	854.4	802.3
75% Percentile	1143	791	1047	1209	985.4
Maximum	1206	952.4	1177	1318	1040
Mean	1032	721.5	906.4	889.1	845.4
Std. Deviation	141.3	130.5	169.7	335.2	134.3
Std. Error of Mean	57.67	53.28	75.89	149.9	67.13
Lower 95% CI	883.8	584.5	695.7	472.8	631.7
Upper 95% CI	1180	858.4	1117	1305	1059
**Captopril – Surrogate Stroke Volume - 1h**					
Treatment (mM)	Ctrl	16	32	64	125
Number of values	6	6	5	5	4
Minimum	0.04	0.02	0.03	0.02	0.03
25% Percentile	0.0475	0.0275	0.035	0.025	0.03
Median	0.055	0.03	0.04	0.04	0.05
75% Percentile	0.0625	0.04029	0.05	0.055	0.05
Maximum	0.07	0.04117	0.06	0.06	0.05
Mean	0.055	0.03186	0.042	0.04	0.04333
Std. Deviation	0.01049	0.007797	0.01095	0.01581	0.01155
Std. Error of Mean	0.004282	0.003183	0.004899	0.007071	0.006667
Lower 95% CI	0.04399	0.02368	0.0284	0.02037	0.01465
Upper 95% CI	0.06601	0.04004	0.0556	0.05963	0.07202

Supplementary Table 6. Descriptive statistics of seven zebrafish cardiovascular endpoints quantified by in vivo imaging after 48h exposure to captopril.

**Captopril – Atrium beat rate - 48h**					
Treatment (mM)	Ctrl	16	32	64	125
Number of values	6	5	6	6	6
Minimum	221.3	216.1	216.2	175.9	175.8
25% Percentile	234.7	219.9	219.8	205.9	197.7
Median	252.1	227.2	223.6	230.6	233.4
75% Percentile	259.6	235.7	230.8	244.9	248.1
Maximum	267.5	239.3	236.9	260.5	250
Mean	248.2	227.7	225	225.5	224.2
Std. Deviation	16.21	8.74	7.229	28.42	29.05
Std. Error of Mean	6.618	3.909	2.951	11.6	11.86
Lower 95% CI	231.2	216.8	217.4	195.7	193.7
Upper 95% CI	265.2	238.5	232.6	255.3	254.7
**Captopril – Ventriculum beat rate - 48h**					
Treatment (mM)	Ctrl	16	32	64	125
Number of values	6	5	6	6	6
Minimum	221.2	216	216.2	175.9	175.8
25% Percentile	234.7	219.9	219.7	205.9	197.7
Median	256.5	232.1	225.1	226.3	233.3
75% Percentile	260.9	245.8	234.2	245.1	249.6
Maximum	267.6	252.3	239.7	261.4	261.4
Mean	249.9	232.7	226.6	224.3	225.7
Std. Deviation	16.82	14.04	8.376	28.68	31.11
Std. Error of Mean	6.869	6.277	3.42	11.71	12.7
Lower 95% CI	232.3	215.3	217.8	194.2	193.1
Upper 95% CI	267.6	250.1	235.3	254.3	258.4
**Captopril – A:V beat ratio - 48h**					
Treatment (mM)	Ctrl	16	32	64	125
Number of values	6	5	6	6	6
Minimum	0.96	0.9	0.95	1	0.95
25% Percentile	0.99	0.95	0.98	1	0.9875
Median	1	1	1	1	1
75% Percentile	1	1	1.005	1.01	1.005
Maximum	1	1	1.02	1.04	1.02
Mean	0.9933	0.98	0.9933	1.007	0.995
Std. Deviation	0.01633	0.04472	0.02338	0.01633	0.02345
Std. Error of Mean	0.006667	0.02	0.009545	0.006667	0.009574
Lower 95% CI	0.9762	0.9245	0.9688	0.9895	0.9704
Upper 95% CI	1.01	1.036	1.018	1.024	1.02
**Captopril – Dorsal Aorta Diameter - 48h**					
Treatment (mM)	Ctrl	16	32	64	125
Number of values	6	5	6	6	6
Minimum	12.2	13.21	11.7	12.26	11.74
25% Percentile	12.49	13.27	12.06	12.61	13.12
Median	12.71	14.49	13.48	12.97	14.02
75% Percentile	13.1	15.13	15.71	14.1	16.01
Maximum	13.98	15.54	17.67	14.73	18.83
Mean	12.83	14.26	13.93	13.26	14.54
Std. Deviation	0.603	0.9837	2.233	0.9019	2.377
Std. Error of Mean	0.2462	0.4399	0.9115	0.3682	0.9706
Lower 95% CI	12.2	13.04	11.58	12.31	12.05
Upper 95% CI	13.46	15.48	16.27	14.2	17.04

Captopril – Average blood flow - 48h					
Treatment (mM)	Ctrl	16	32	64	125
Number of values	6	5	6	6	6
Minimum	0.15	0.15	0.14	0.09	0.18
25% Percentile	0.1875	0.175	0.14	0.1275	0.1875
Median	0.28	0.2	0.165	0.165	0.215
75% Percentile	0.3225	0.255	0.215	0.2475	0.25
Maximum	0.36	0.28	0.29	0.3	0.28
Mean	0.2633	0.212	0.1817	0.1817	0.22
Std. Deviation	0.07633	0.04764	0.05636	0.0736	0.03742
Std. Error of Mean	0.03116	0.02131	0.02301	0.03005	0.01528
Lower 95% CI	0.1832	0.1528	0.1225	0.1044	0.1807
Upper 95% CI	0.3434	0.2712	0.2408	0.2589	0.2593
**Captopril – Linear blood flow - 48h**					
Treatment (mM)	Ctrl	16	32	64	125
Number of values	6	5	6	6	6
Minimum	1010	870.2	693	725.3	822
25% Percentile	1299	888.4	868.7	799.1	842.9
Median	1454	1042	987.4	1011	896.6
75% Percentile	1581	1251	1263	1176	1077
Maximum	1622	1348	1385	1290	1282
Mean	1417	1064	1034	999.8	959.2
Std. Deviation	215.8	194.5	243.2	205.6	172.3
Std. Error of Mean	88.11	86.99	99.29	83.95	70.33
Lower 95% CI	1190	822.7	778.4	784	778.4
Upper 95% CI	1643	1306	1289	1216	1140
**Captopril – Surrogate Stroke Volume - 48h**					
Treatment (mM)	Ctrl	16	32	64	125
Number of values	6	5	6	6	6
Minimum	0.04	0.04	0.04	0.02	0.04
25% Percentile	0.04	0.045	0.04	0.035	0.0475
Median	0.07	0.05	0.04	0.05	0.055
75% Percentile	0.0725	0.065	0.055	0.065	0.07
Maximum	0.08	0.07	0.07	0.08	0.1
Mean	0.06167	0.054	0.04667	0.05	0.06
Std. Deviation	0.01722	0.0114	0.01211	0.02	0.02098
Std. Error of Mean	0.007032	0.005099	0.004944	0.008165	0.008563
Lower 95% CI	0.04359	0.03984	0.03396	0.02901	0.03799
Upper 95% CI	0.07974	0.06816	0.05938	0.07099	0.08201

Supplementary Table 7. The zebrafish cardiovascular assay described in this study was performed using a sample size equal to 6 fish per treatment group. This table displays the estimated minimum effect size (%) (i.e. the minimum difference between two groups) detected with n=6 for each drug/endpoint/time combination. These values were calculated using endpoint-specific mean and standard deviation observed in the control populations (Column 1-6), with acceptable power set at 0.80 and significance level (alpha) set at 0.05 (two-sided test). As term of comparison, Column 7 displays the estimated effect size detected with n=6 calculated using data retrieved from Parker et al. (2014).

		Estimated minimum effect size (%) detected with n=6 (power=0.80, alpha=0.05)						
		**1**	**2**	**3**	**4**	**5**	**6**	**7**
		PRO 1h	LOS 1h	CAP 1h	PRO 48h	LOS 48h	CAP 48h	Parker et al. 2014
**Endpoint**	ABR	13	4	12	9	15	12	11
	VBR	13	4	7	9	15	12	13
	A:V beat ratio	1.8	0.9	7	1	1	3	10
	DA diameter	19	20	5	8	12	9	24
	DA average BF	26	47	42	40	32	52	22
	DA linear BF	23	18	25	20	14	25	n/a
	SSV	47	47	35	7	35	28	43

PRO: propranolol; LOS: losartan; CAP: captopril; 1h: 1 hour exposure; 48h: 48 hours exposure; ABR: atrial beat rate per minute; VBR: ventricular beat rate per minute; A:V: atrium:ventriculum; DA: dorsal aorta; BF: blood flow; SSV: surrogate stroke volume.

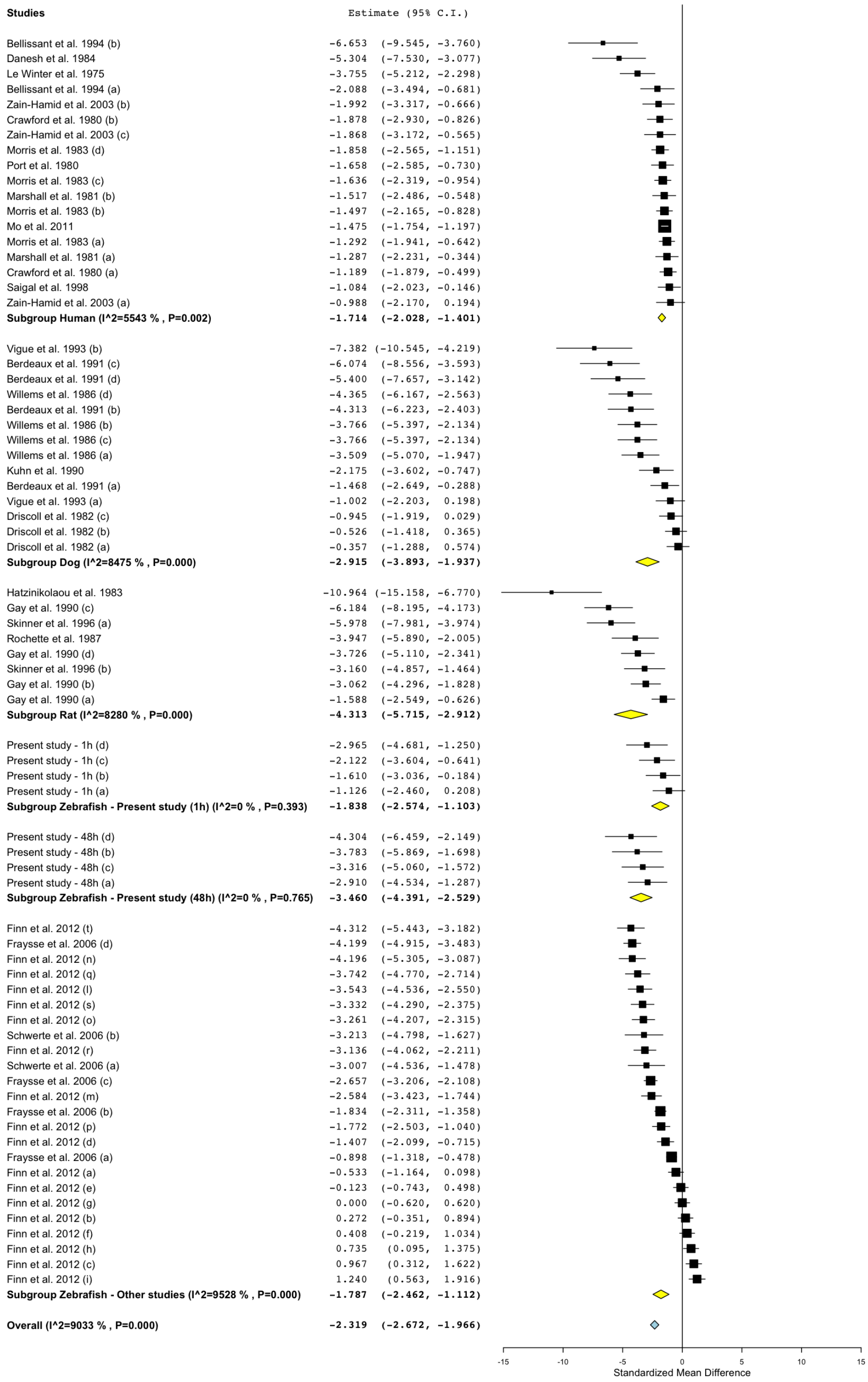

Supplementary Figure 1. Meta-analysis of the effects of propranolol on heart-rate in zebrafish, rat, dog, and humans.

Studies	Estimate (95% C.I.)
Bellissant et al. 1994 (a)	-1.167 (-2.391, 0.057)
Saigal et al. 1998	-0.282 (-1.163, 0.599)
Zain-Hamid et al. 2003 (c)	-0.119 (-1.251, 1.014)
Zain-Hamid et al. 2003 (a)	0.323 (-0.814, 1.460)
Zain-Hamid et al. 2003 (b)	0.485 (-0.659, 1.629)
Bellissant et al. 1994 (b)	0.923 (-0.268, 2.113)
**Subgroup Human (I^2=3090 % , P=0.204)**	**0.017 (-0.526, 0.561)**
Vigue et al. 1993 (b)	-3.279 (-5.011, -1.547)
Berdeaux et al. 1991 (d)	-1.547 (-2.741, -0.353)
Berdeaux et al. 1991 (c)	-1.430 (-2.604, -0.256)
Berdeaux et al. 1991 (b)	-1.133 (-2.261, -0.004)
Vigue et al. 1993 (a)	-1.124 (-2.342, 0.093)
Berdeaux et al. 1991 (a)	-0.485 (-1.548, 0.578)
Kuhn et al. 1990	0.000 (-1.132, 1.132)
**Subgroup Dog (I^2=4930 % , P=0.066)**	**-1.157 (-1.800, -0.515)**
Chillon & Baumbach 1998 (b)	3.888 (2.648, 5.127)
Chillon & Baumbach 1998 (a)	4.860 (3.411, 6.309)
**Subgroup Rat (I^2=0 % , P=0.318)**	**4.298 (3.356, 5.240)**
Present study - 1h (a)	-0.330 (-1.469, 0.809)
Present study - 1h (b)	-0.235 (-1.425, 0.956)
Present study - 1h (c)	0.242 (-0.894, 1.377)
Present study - 1h (d)	1.037 (-0.168, 2.242)
**Subgroup Zebrafish - 1h (I^2=587 % , P=0.364)**	**0.165 (-0.437, 0.766)**
Present study - 48h (d)	-1.404 (-2.728, -0.080)
Present study - 48h (a)	-0.178 (-1.312, 0.956)
Present study - 48h (c)	-0.162 (-1.296, 0.971)
Present study - 48h (b)	-0.023 (-1.155, 1.109)
**Subgroup Zebrafish - 48h (I^2=0 % , P=0.401)**	**-0.373 (-0.959, 0.214)**
**Overall (I^2=8305 % , P=0.000)**	**-0.045 (-0.640, 0.550)**

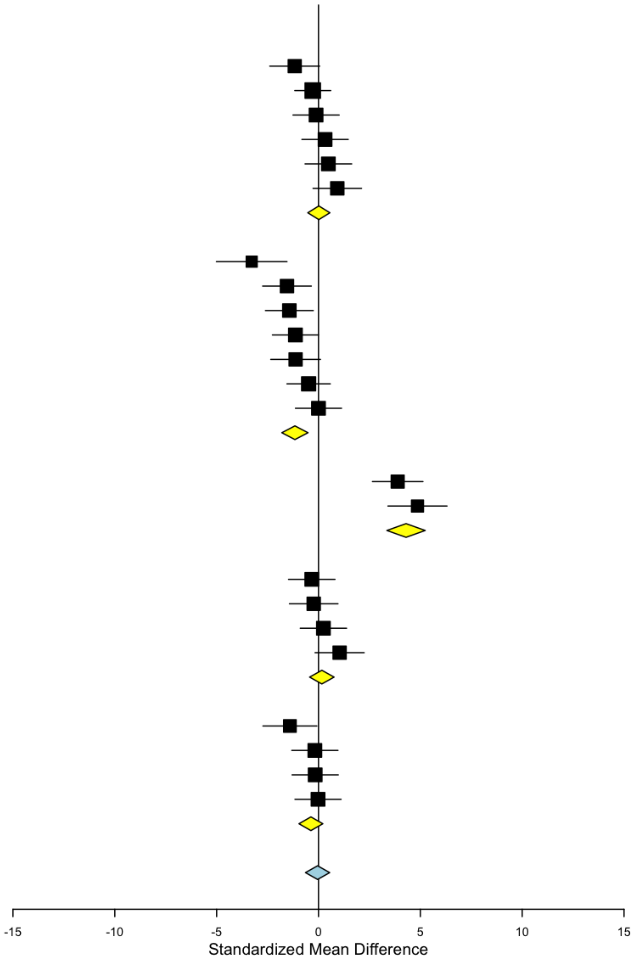

Supplementary Figure 2. Meta-analysis of the effects of propranolol on vessel diameter in zebrafish, rat, dog, and humans.

Studies	Estimate (95% C.I.)
Bellissant et al. 1994 (a)	-2.214 (-3.651, -0.777)
Port et al. 1980	-0.038 (-0.839, 0.762)
Mo et al. 2011	0.197 (-0.050, 0.445)
Bellissant et al. 1994 (b)	1.628 (0.322, 2.933)
**Subgroup Human (I^2=8084 % , P=0.001)**	**-0.031 (-0.982, 0.919)**
Driscoll et al. 1982 (b)	-0.867 (-1.784, 0.050)
Driscoll et al. 1982 (a)	-0.317 (-1.247, 0.612)
Driscoll et al. 1982 (c)	-0.190 (-1.116, 0.736)
Kuhn et al. 1990	1.105 (-0.110, 2.320)
**Subgroup Dog (I^2=5403 % , P=0.089)**	**-0.143 (-0.870, 0.585)**
Hatzinikolaou et al. 1983	-3.848 (-5.616, -2.079)
Rochette et al. 1987	-1.603 (-2.904, -0.303)
Gay et al. 1990 (b)	1.851 (0.850, 2.853)
Gay et al. 1990 (a)	2.405 (1.305, 3.504)
**Subgroup Rat (I^2=9419 % , P=0.000)**	**-0.226 (-2.805, 2.353)**
Present study - 1h (a)	-0.262 (-1.454, 0.930)
Present study - 1h (b)	0.612 (-0.602, 1.826)
Present study - 1h (c)	0.700 (-0.465, 1.866)
Present study - 1h (d)	2.882 (1.267, 4.498)
**Subgroup Zebrafish - 1h (I^2=6848 % , P=0.023)**	**0.887 (-0.254, 2.027)**
Present study - 48h (a)	-0.449 (-1.595, 0.697)
Present study - 48h (d)	-0.082 (-1.348, 1.184)
Present study - 48h (b)	0.159 (-1.030, 1.348)
Present study - 48h (c)	0.208 (-0.926, 1.343)
**Subgroup Zebrafish - 48h (I^2=0 % , P=0.853)**	**-0.041 (-0.632, 0.549)**
**Overall (I^2=7936 % , P=0.000)**	**0.134 (-0.345, 0.613)**

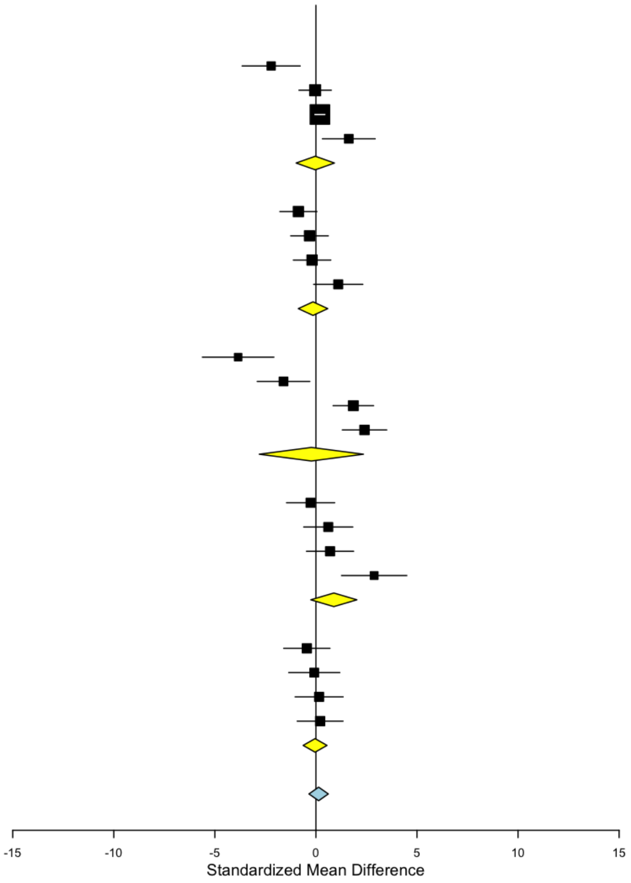

Supplementary Figure 3. Meta-analysis of the effects of propranolol on stroke volume in zebrafish, rat, dog, and humans.

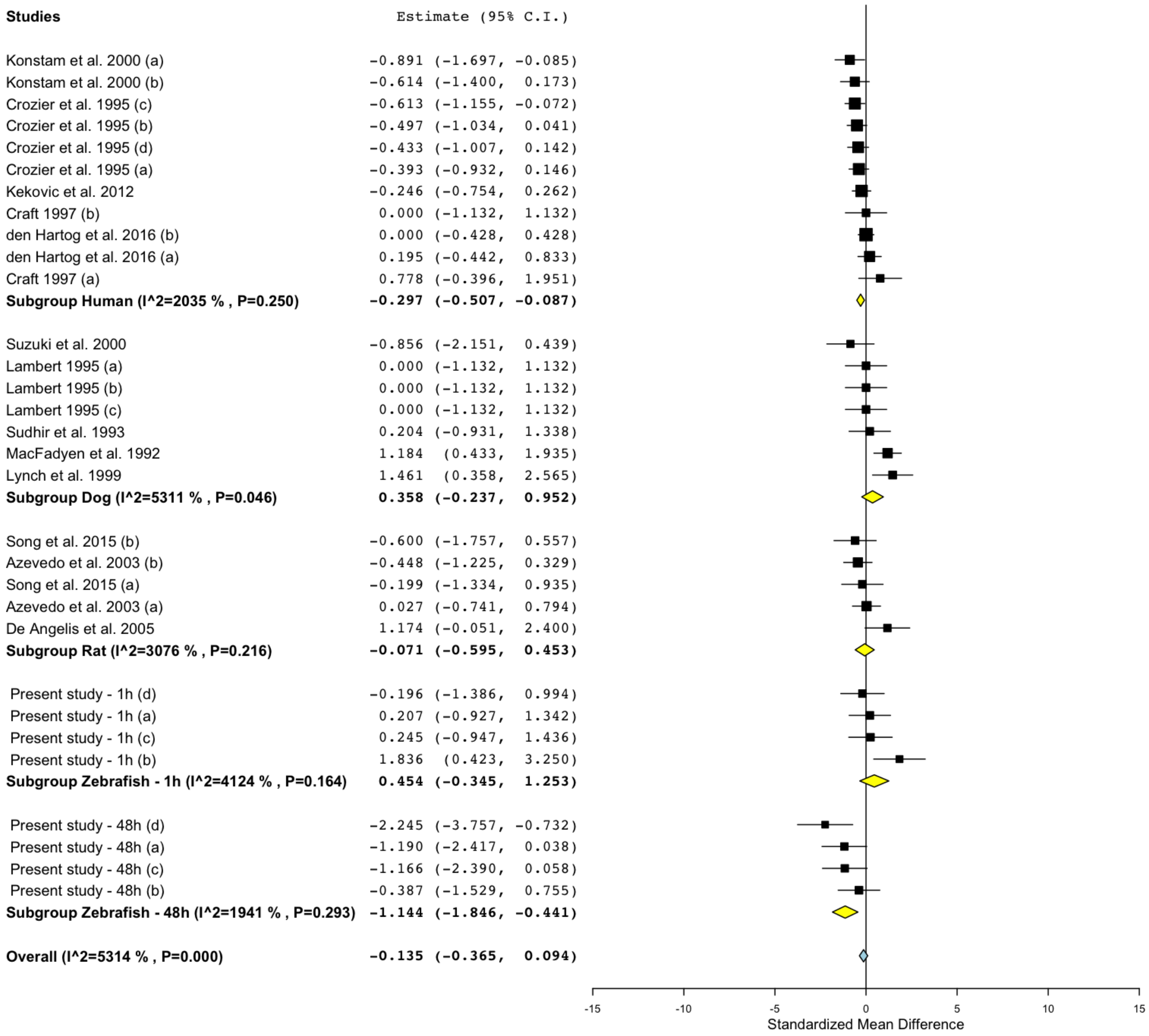

Supplementary Figure 4. Meta-analysis of the effects of losartan on heart rate in zebrafish, rat, dog, and humans.

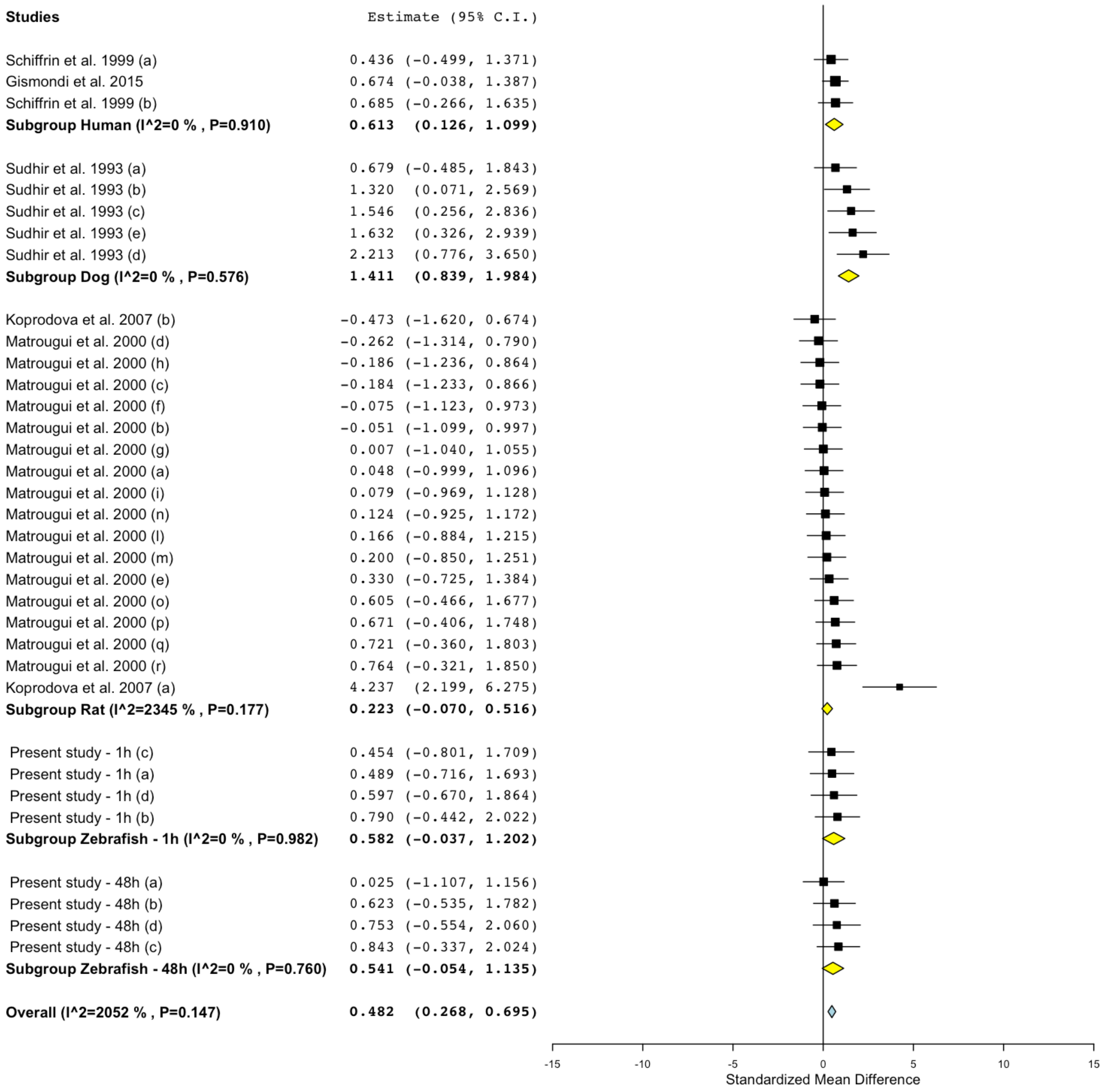

Supplementary Figure 5. Meta-analysis of the effects of losartan on vessel diameter in zebrafish, rat, dog, and humans.

Studies	Estimate (95% C.I.)
Craft 1997 (a)	-0.539 (-1.691, 0.613)
den Hartog et al. 2016 (b)	-0.082 (-0.510, 0.346)
Craft 1997 (b)	0.000 (-1.132, 1.132)
den Hartog et al. 2016 (a)	0.651 (-0.001, 1.304)
**Subgroup Human (I^2=3508 % , P=0.202)**	**0.084 (-0.376, 0.545)**
Suzuki et al. 2000	0.995 (-0.319, 2.309)
**Subgroup Dog (I^2=NA , P=NA)**	**0.995 (-0.319, 2.309)**
Azevedo et al. 2003 (b)	0.000 (-0.768, 0.768)
Azevedo et al. 2003 (a)	0.485 (-0.294, 1.263)
De Angelis et al. 2005	0.923 (-0.268, 2.113)
**Subgroup Rat (I^2=0 % , P=0.406)**	**0.358 (-0.139, 0.855)**
Present study - 1h (a)	0.316 (-0.878, 1.510)
Present study - 1h (d)	0.436 (-0.818, 1.691)
Present study - 1h (b)	0.609 (-0.605, 1.823)
Present study - 1h (c)	0.961 (-0.348, 2.270)
**Subgroup Zebrafish - 1h (I^2=0 % , P=0.905)**	**0.567 (-0.054, 1.187)**
Present study - 48h (c)	-0.142 (-1.275, 0.991)
Present study - 48h (b)	0.068 (-1.064, 1.200)
Present study - 48h (d)	1.061 (-0.206, 2.328)
Present study - 48h (a)	1.486 (0.208, 2.764)
**Subgroup Zebrafish - 48h (I^2=3766 % , P=0.186)**	**0.570 (-0.189, 1.330)**
**Overall (I^2=696 % , P=0.374)**	**0.318 (0.075, 0.561)**

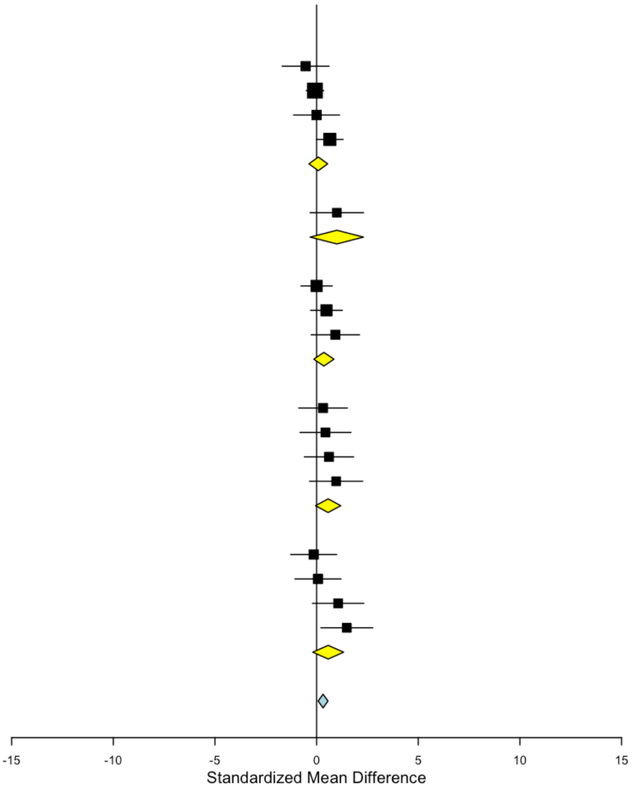

Supplementary Figure 6. Meta-analysis of the effects of losartan on stroke volume in zebrafish, rat, dog, and humans.

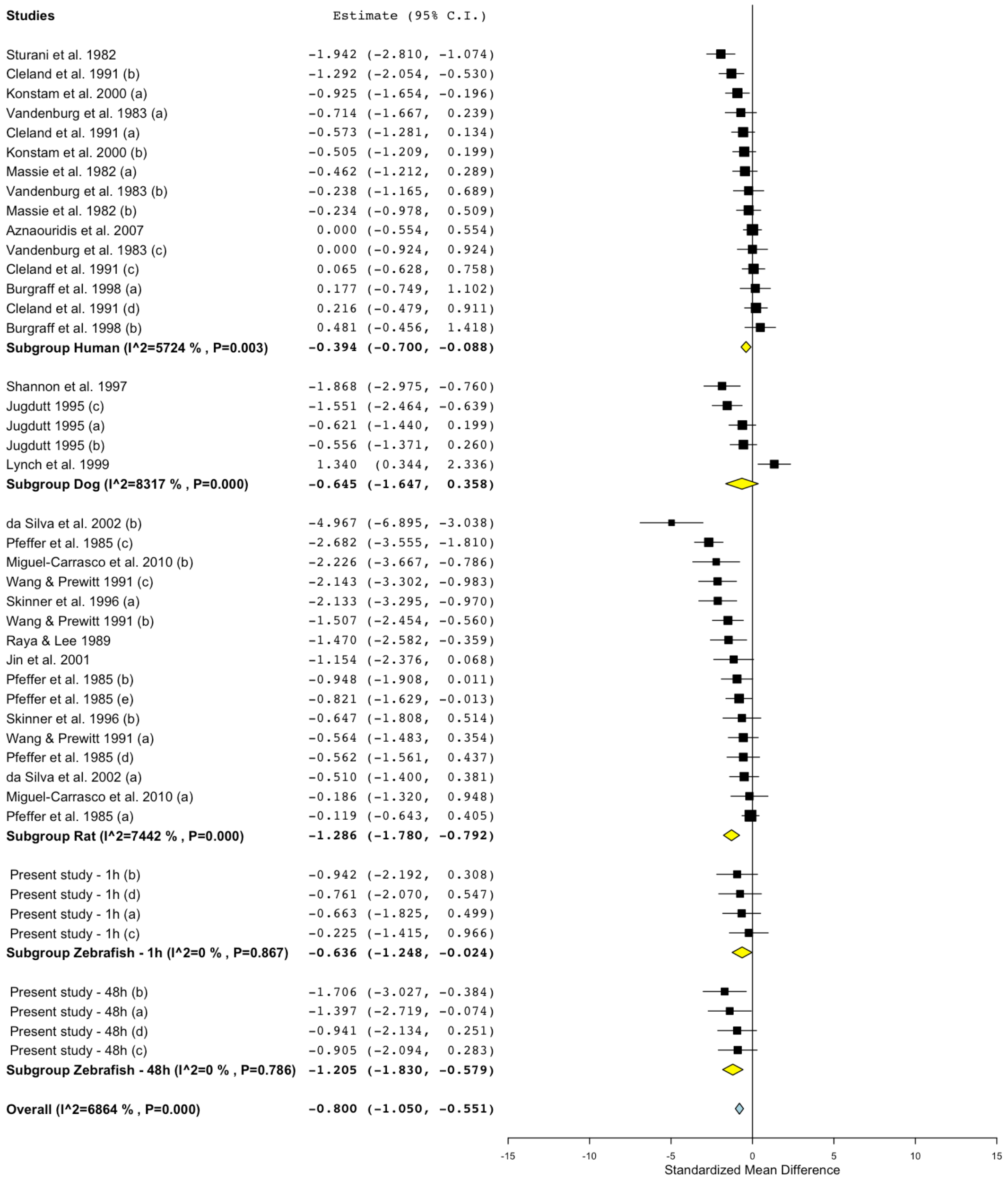

Supplementary Figure 7. Meta-analysis of the effects of captopril on heart rate in zebrafish, rat, dog, and humans.

Studies	Estimate (95% C.I.)
Aznaouridis et al. 2007	0.007 (-0.548, 0.561)
Chau et al. 1992	2.857 (1.464, 4.249)
**Subgroup Human (I^2=9280 % , P=0.000)**	**1.357 (-1.432, 4.146)**
Wang & Prewitt 1991 (c)	-0.497 (-1.435, 0.441)
Freslon & Giudicelli 1983 (a)	0.238 (-0.642, 1.117)
Wang & Prewitt 1991 (a)	0.286 (-0.619, 1.191)
Wang & Prewitt 1991 (b)	1.096 (0.200, 1.992)
Kimura et al. 1991 (b)	3.098 (1.260, 4.937)
Freslon & Giudicelli 1983 (b)	3.312 (1.963, 4.662)
Kimura et al. 1991 (c)	3.778 (1.803, 5.752)
Kimura et al. 1991 (a)	3.974 (1.836, 6.112)
Kimura et al. 1991 (d)	5.522 (2.927, 8.116)
**Subgroup Rat (I^2=8619 % , P=0.000)**	**2.030 (0.891, 3.169)**
Present study - 1h (c)	-0.513 (-1.798, 0.772)
Present study - 1h (b)	-0.034 (-1.221, 1.153)
Present study - 1h (a)	0.505 (-0.645, 1.654)
Present study - 1h (d)	1.416 (0.007, 2.825)
**Subgroup Zebrafish - 1h (I^2=3139 % , P=0.224)**	**0.307 (-0.448, 1.062)**
Present study - 48h (c)	0.517 (-0.633, 1.668)
Present study - 48h (b)	0.621 (-0.538, 1.779)
Present study - 48h (d)	0.910 (-0.279, 2.099)
Present study - 48h (a)	1.643 (0.272, 3.015)
**Subgroup Zebrafish - 48h (I^2=0 % , P=0.622)**	**0.865 (0.261, 1.469)**
**Overall (I^2=7871 % , P=0.000)**	**1.248 (0.664, 1.831)**

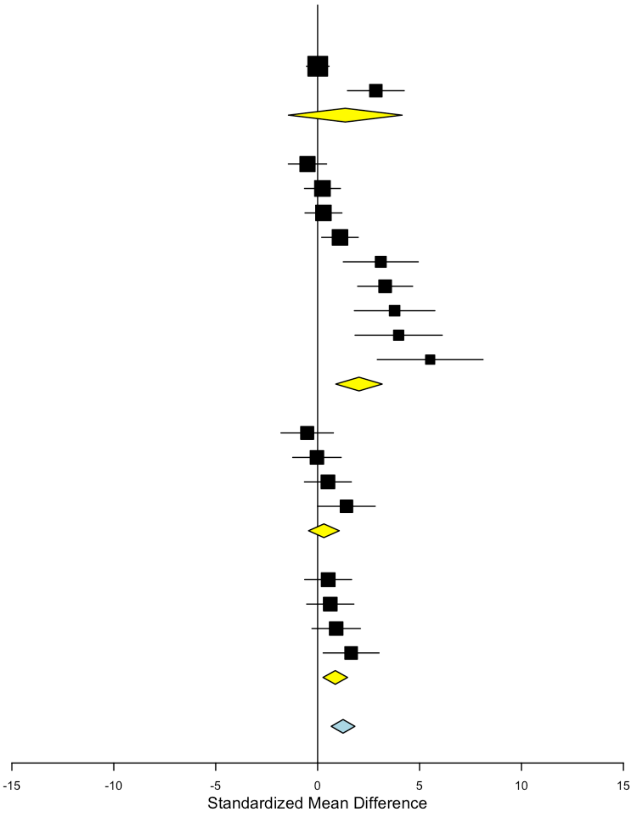

Supplementary Figure 8. Meta-analysis of the effects of captopril on vessel diameter in zebrafish, rat, dog, and humans.

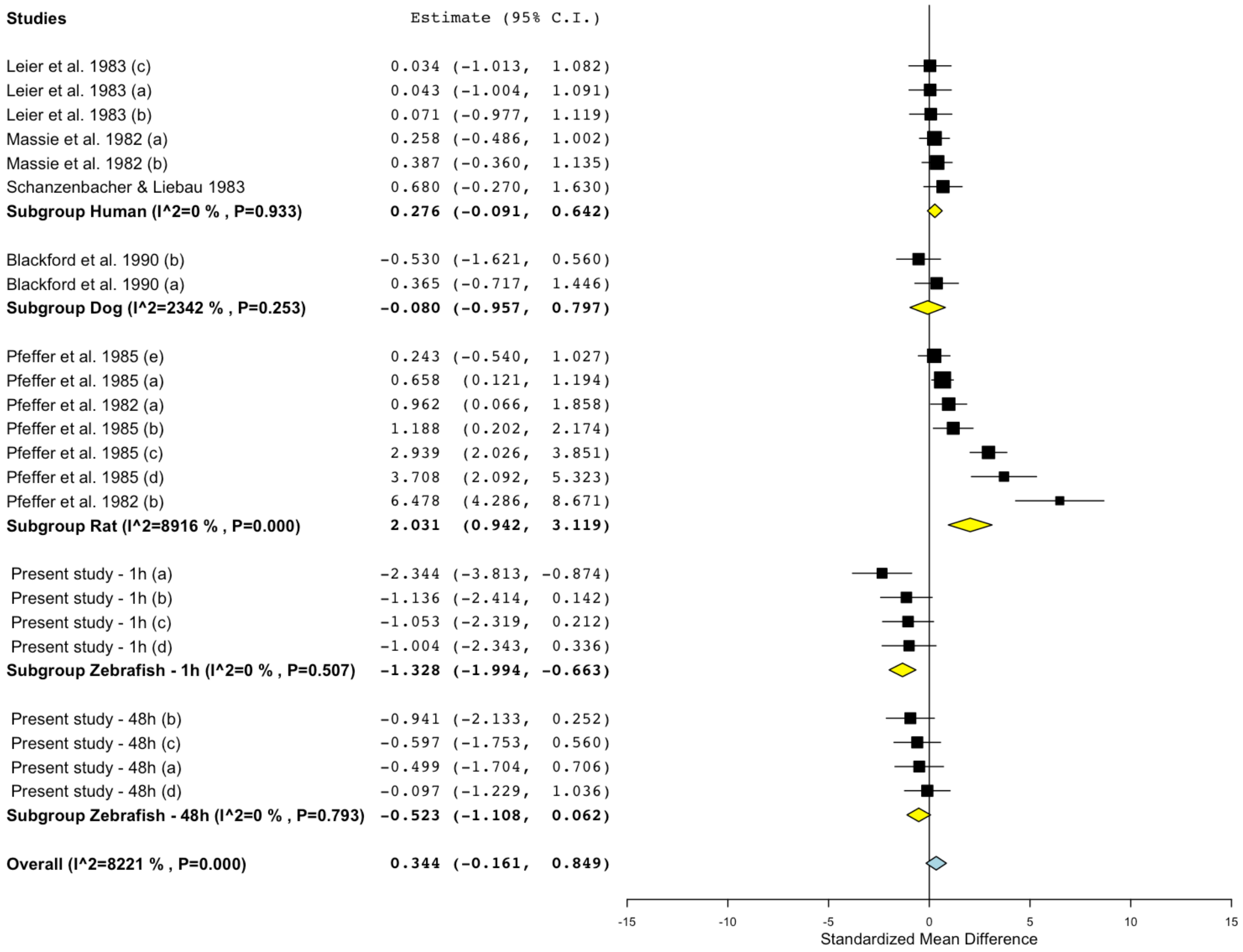

Supplementary Figure 9. Meta-analysis of the effects of captopril on stroke volume in zebrafish, rat, dog, and humans.
